# Isocitrate Dehydrogenase Mutations in Cancer: From Bench to Bedside Applications

**DOI:** 10.1002/mco2.70732

**Published:** 2026-04-19

**Authors:** Yuhan Fang, Xiaoqing Wang, Kai Luo, Tikam Chand Dakal, He Bai, Caiming Xu, Guixin Zhang

**Affiliations:** ^1^ Department of General Surgery The Second Hospital of Dalian Medical University Dalian China; ^2^ Institute (College) of Integrative Medicine Dalian Medical University Dalian China; ^3^ Laboratory of Integrative Medicine The First Affiliated Hospital of Dalian Medical University Dalian China; ^4^ Department of Biotechnology Mohanlal Sukhadia University Udaipur Rajasthan India; ^5^ Beckman Research Institute of City of Hope Biomedical Research Center Monrovia California USA

**Keywords:** 2‐hydroxyglutarate, isocitrate dehydrogenase mutation, targeted inhibitors, therapeutic resistance, tumor immune microenvironment

## Abstract

Isocitrate dehydrogenase (IDH) mutations represent pivotal oncogenic drivers across multiple malignancies. Mutant IDH enzymes acquire neomorphic activity that produces the oncometabolite d‐2‐hydroxyglutarate (D‐2HG), which competitively inhibits α‐ketoglutarate‐dependent dioxygenases and promotes epigenetic reprogramming, differentiation arrest, and malignant transformation. Beyond tumor cell–intrinsic effects, D‐2HG profoundly remodels the tumor immune microenvironment by directly suppressing T‐cell proliferation and effector functions, silencing natural killer (NK) cell‐activating ligands, and impairing dendritic cell maturation. In this review, we delineate the mechanistic basis of mutant IDH in oncogenesis and evaluate the development of selective allosteric inhibitors validated through rigorous preclinical models demonstrating potent D‐2HG suppression. Clinical translation has yielded multiple FDA‐approved IDH inhibitors demonstrating significant therapeutic efficacy across diverse IDH‐mutant malignancies. Notably, dual inhibitors have extended progression‐free survival in gliomas, whereas triple‐combination regimens have achieved substantial complete remission rates in acute myeloid leukemia. However, therapeutic resistance has emerged through second‐site mutations, clonal evolution, and metabolic reprogramming. We also discuss rational combinatorial strategies integrating IDH inhibitors with hypomethylating agents (HMAs), targeted therapies, and immunomodulatory approaches, alongside emerging technologies such as single‐cell profiling and spatial transcriptomics. By addressing both achievements and challenges, this review underscores the translational relevance of IDH‐targeted therapy and its potential to reshape precision oncology through refined patient stratification and enhanced therapeutic efficacy.

## Graphical abstract

Mutant isocitrate dehydrogenase affects multiple cancer types. Alterations in IDH1 and IDH2 result in abnormal enzyme activity, leading to the overproduction of D‐2‐hydroxyglutarate (D‐2HG). This metabolite disrupts cellular metabolism and epigenetic regulation, driving cancer initiation, progression, and metastasis. Consequently, IDH has emerged as a key pharmacological target, spurring the development of a novel class of targeted therapies for IDH1‐ and IDH2‐mutant malignancies. However, resistance mechanisms to mIDH inhibitors, including secondary mutations, epigenetic reprogramming, and metabolic adaptations, pose significant challenges to their efficacy. This review explores the prognostic and therapeutic potential of mIDH, addresses emerging resistance mechanisms, and highlights its role in advancing precision medicine and enhancing patient outcomes.

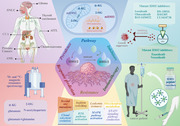

## 1 Introduction

Mutations in the genes encoding the cytoplasmic and mitochondrial forms of isocitrate dehydrogenase (IDH) are frequently detected in various malignancies, with IDH1 (R132) and IDH2 (R140/R172) representing the most common metabolic gene mutations in cancer. Since 2008, when IDH1 mutations were first identified as critical pathogenic drivers in glioblastoma multiforme (GBM) [1], subsequent research has uncovered IDH mutations (mIDH) across a broad spectrum of cancers, including acute myeloid leukemia (AML), cholangiocarcinoma (CCA), and central and periosteal chondrosarcoma [2, 3, 4, 5, 6, 7]. As integral components of the citric acid cycle, the IDH family comprises three isoenzymes: the NADP^+^‐dependent IDH1 and IDH2 and the NAD^+^‐dependent IDH3 [8, 9]. IDH1 and IDH2 can reversibly catalyze the reductive carboxylation of α‐ketoglutarate (the reverse reaction) under specific conditions such as hypoxia, whereas the IDH3‐catalyzed reaction is essentially irreversible due to thermodynamic constraints. Unique among metabolic enzymes for their profound influence on tumor initiation and progression, mutated IDH1 and IDH2 confer neomorphic activity that leads to the aberrant generation of high levels of the oncometabolite (*R*)‐2‐hydroxyglutarate (2‐HG) [9, 10, 11]. This metabolite competitively inhibits α‐ketoglutarate (α‐KG)‐dependent dioxygenases, thereby perturbing epigenetic regulation, cellular metabolism, DNA repair, and differentiation programs that collectively facilitate malignant transformation [12, 13, 14].

Over the past decade, the discovery of mIDH has catalyzed novel therapeutic development, with small‐molecule allosteric inhibitors achieving the most advanced clinical translation. Preclinical and clinical studies have demonstrated that targeting mIDH can alleviate the downstream metabolic and epigenomic consequences of pathological D‐2HG accumulation and translate into meaningful clinical benefit [15]. In 2017, the FDA approved the first mIDH2 inhibitor, enasidenib [16]. This was followed by approval of the mIDH1 inhibitors ivosidenib and olutasidenib, as well as the dual mIDH1/2 inhibitor vorasidenib [17, 18, 19, 20, 21, 22]. Other targeted agents (e.g., HMPL‐306 and TQB3454) are currently undergoing clinical evaluation [23]. Inhibition of mutant IDH first demonstrated value in the treatment of hematological malignancies and has since advanced further in gliomas and other solid tumors [8]. Mutant IDH inhibitors can be administered as monotherapy or in combination with targeted agents against other oncogenic pathways, including HMAs (e.g., azacitidine [AZA]), immune checkpoint inhibitors, and pathway‐targeted therapies such as B‐cell lymphoma 2 (BCL‐2) or Janus kinase 2 (JAK2) inhibitors, to further enhance therapeutic efficacy [24, 25, 26, 27, 28, 29].

Despite these advances, resistance and relapse remain common and limit the durability of responses to mIDH‐targeted therapy. Mechanistically, loss of sensitivity can result from tumor‐intrinsic alterations (e.g., resistance‐associated variants and clonal evolution and resistance‐related pathways activation) [30, 31, 32, 33, 34, 35, 36]. Second‐site mutations in IDH1 or IDH2 increase D‐2HG production and alter inhibitor binding sites, thereby conferring acquired resistance [37]. Similarly, isoform switching—whereby IDH1‐mutant tumors become dependent on IDH2, or IDH2‐mutant tumors rely on IDH1—sustains continuous D‐2HG synthesis and reduces the efficacy of single‐isoform inhibitors [38, 39]. Clonal selection during therapy further exacerbates resistance, with subclones harboring mutations in genes such as FMS‐like tyrosine kinase 3 (FLT3), neuroblastoma RAS viral oncogene homolog (NRAS), and runt‐related transcription factor 1 (RUNX1) expanding under drug‐selective pressure [39]. Additionally, enhanced leukemia stem cell (LSC) characteristics, marked by upregulation of FOXC1 and CD99 expression, are closely associated with primary resistance and diminished therapeutic responses [39]. Nongenetic mechanisms also play critical roles: Mitochondrial metabolic reprogramming, including enhanced oxidative phosphorylation (OXPHOS) and fatty acid β‐oxidation (FAO), enables cellular survival in the presence of mIDH inhibitors [40]; meanwhile, aberrant activation of mitogen‐activated protein kinase (MAPK), receptor tyrosine kinase (RTK), and signal transducer and activator of transcription 5 (STAT5) signaling pathways bypasses the differentiation blockade mediated by mIDH inhibitors, ultimately leading to primary or acquired resistance [30, 31, 32]. Together, these challenges underscore the need for next‐generation mIDH inhibitors and rational combinations, guided by molecular profiling to refine patient stratification and to enable biomarker‐informed treatment optimization within precision oncology.

In this review, we systematically summarize the translational trajectory of IDH‐targeted therapy from bench to bedside. We first elucidate the molecular mechanisms by which mIDH drives oncogenesis through D‐2HG‐mediated epigenetic reprogramming and immune microenvironment remodeling. We then discuss preclinical advances in inhibitor development and validation, followed by the clinical translation and efficacy of FDA‐approved agents. Finally, we analyze resistance mechanisms and rational combination strategies designed to overcome current therapeutic limitations while evaluating biomarker‐guided approaches for monitoring treatment response. By integrating current achievements and unresolved challenges, this review aims to provide comprehensive insights into IDH‐targeted precision oncology and guide future therapeutic innovation.

## 2 Bench Discovery: Molecular Mechanisms and Cellular Metabolism of mIDH

Numerous studies have identified mIDH as frequent initiators and drivers of oncogenesis across several tumor types (Table 1). These mutations confer neomorphic enzymatic activity that generates the oncometabolite D‐2HG, which profoundly alters cellular biology and contributes to tumor progression through metabolic reprogramming, epigenetic modifications, and differentiation blockade. Multi‐region sequencing has revealed substantial intratumoral heterogeneity in various cancers, demonstrating that mIDH reshape the tumor microenvironment (TME) at multiple levels, thereby influencing both disease progression and treatment response.

**TABLE 1:** Therapeutic implications of IDH1 and IDH2 mutations in different cancers.

Cancer type	IDH mutation type	Prevalence (%)	Impact on tumor growth	Impact on metastasis	Potential therapeutic target	Reference
Glioblastoma multiforme (GBM)	IDH1 (R132)	11.3–12	Impairs cellular metabolism, reduces tumor differentiation potential	IDH1 mutation correlates with higher survival but increased local invasion	IDH1‐targeted therapies (e.g., Ivosidenib), with epigenetic or metabolic targets	[1, 41, 42]
Acute myeloid leukemia (AML)	IDH1 (R132), IDH2 (R140, R172)	9.2–20	Promotes abnormal proliferation and differentiation of hematopoietic cells	IDH2 R140 mutation is associated with better prognosis and reduced metastasis in young AML patients	IDH1/IDH2 inhibitors (e.g., enasidenib and ivosidenib)	[2, 3, 4, 43]
Cholangiocarcinoma (CCA)	IDH1 (R132), IDH2 (R140, R172)	13.9–23	Leads to DNA hypermethylation, disrupting gene expression	Promotes angiogenesis and tumor spread via VEGF pathways	IDH1‐targeting drugs (e.g., ivosidenib), VEGF inhibitors	[5, 44]
Bone and cartilage tumors	IDH1 (R132), IDH2 (R140, R172)	40–56	Stimulates cartilage tumorigenesis by affecting histone methylation	Increases tumor aggressiveness, causes local metastasis	Potential use of IDH inhibitors, exploring epigenetic therapies	[6, 7, 45]
Gliomas	IDH1 (R132), IDH2 (R140, R172)	16–88	Enhance tumor growth by blocking cell differentiation and disrupting epigenetic regulation	D‐2HG accumulation leads to reduced cell differentiation, increased invasiveness	IDH inhibitors (e.g., ivosidenib), targeting D‐2HG accumulation	[46, 47]
Prostate cancer	IDH1 (R132)	2.7	Promotes progression through epigenetic alterations	Rarely linked to significant metastasis	IDH inhibitors may be considered, though rare in prostate cancer	[46]
Sinonasal undifferentiated carcinoma (SNUC)	IDH2 (R172)	14.4–36.2	Promotes rapid proliferation and resistance to treatment	Increases potential for local invasion and metastasis	IDH2 inhibitors, combined treatment targeting resistant pathways	[48, 49, 50]
Angioimmunoblastic T‐cell lymphoma (AITL)	IDH2 (R172)	20–45	Causes immune suppression and enhances abnormal lymphoid growth	Higher potential for systemic spread	IDH2‐targeted therapies, immunomodulatory treatments	[51, 52]
Thyroid carcinoma	IDH1 (R132)	7.89–16	Alters metabolic pathways, leading to aggressive tumor growth	Increased metastatic risk, though relatively uncommon	IDH inhibitors, experimental approaches targeting metabolic pathways	[53, 54]
Pediatric AML	IDH1 (R132), IDH2 (R140, R172)	3.4	Alters developmental pathways of hematopoietic cells	Lower rate of metastasis compared to adult AML	IDH inhibitors, focusing on early intervention in younger patients	[43]

Abbreviations: D‐2HG, d‐2‐hydroxyglutarate; IDH, isocitrate dehydrogenase; VEGF, vascular endothelial growth factor.

### 2.1 Biological Oncogenesis of mIDH

**FIGURE 1:**
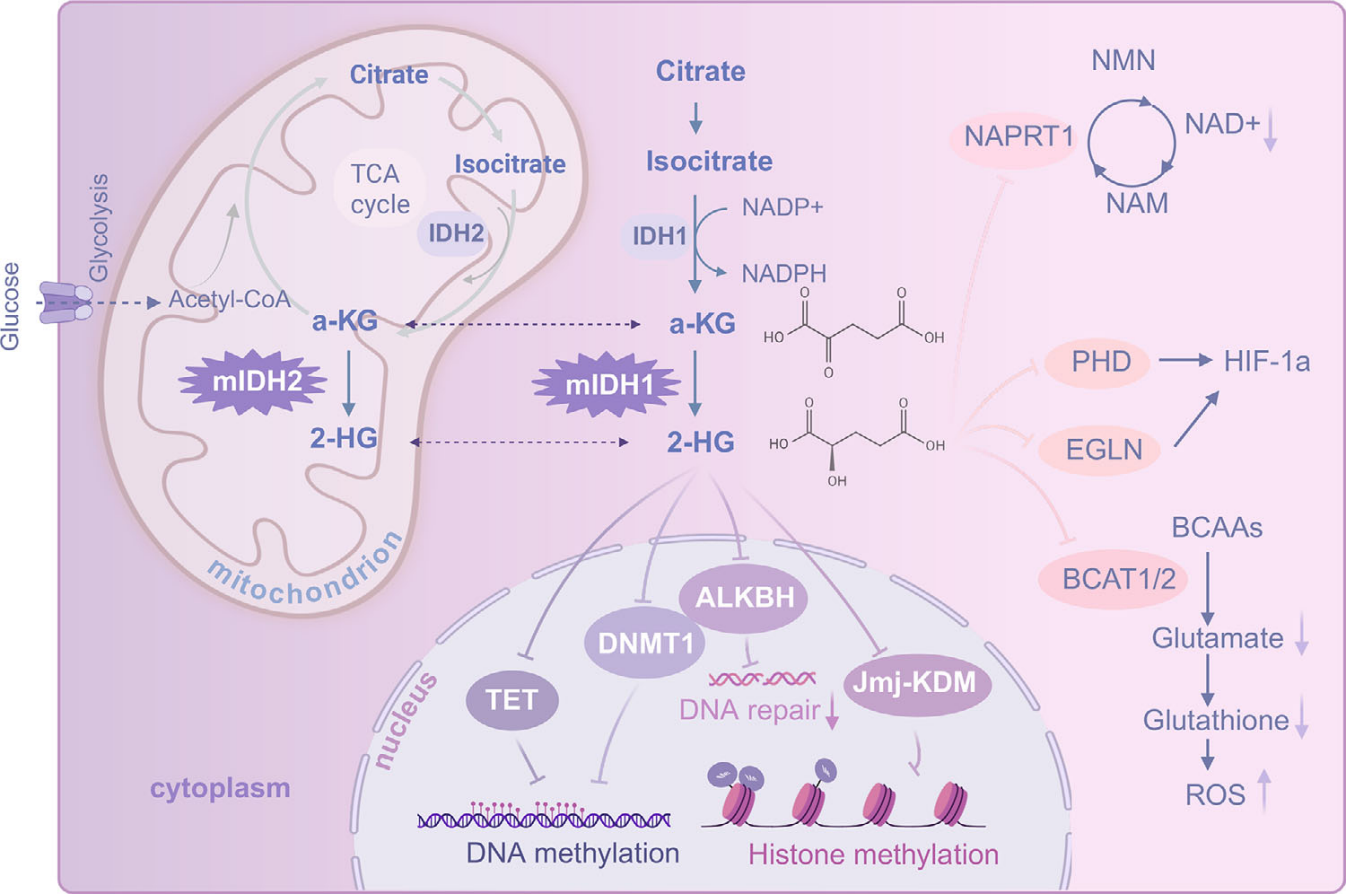
Molecular mechanisms of IDH mutations in tumors. IDH1 resides in the cytoplasm and peroxisomes, whereas IDH2 is found in the mitochondrial matrix. IDH1/2 convert isocitrate into α‐ketoglutarate (α‐KG) and CO_2_, generating NADPH. Mutations in IDH1 and IDH2 generate neomorphic enzymatic activity, resulting in excessive synthesis of D‐2‐hydroxyglutarate (D‐2HG). D‐2HG competes with α‐KG and inhibits enzymes such as KDMs, TETs, and DNMT1, leading to increased DNA and histone methylation. It also hinders DNA repair by blocking α‐KG‐dependent hydroxylase homologs (ALKBH). Mutations influence hypoxia‐inducible factor (HIF) expression through interactions with the PHD and EGLN. D‐2HG also disturbs cellular homeostasis by suppressing branched‐chain amino acid transaminases (BCAT1/2) and nicotinic acid phosphoribosyl transferase 1 (NAPRT1). BCAA, branched‐chain amino acid; IDH, isocitrate dehydrogenase; PHD, prolyl hydroxylase domain; ROS, reactive oxygen species; TCA, tricarboxylic acid; TET, ten‐eleven translocation.

The IDH family consists of three isozymes, IDH1, IDH2, and IDH3, which possess distinct structural characteristics (Figure 1). These enzymes play a vital role in the tricarboxylic acid (TCA) cycle, catalyzing the oxidative decarboxylation of isocitrate into α‐KG and CO_2_ [55]. IDH1 and IDH2 are highly similar homodimers. Each dimeric subunit contains three structural domains: a large domain with a typical Rossmann fold, a clasp domain forming two antiparallel β‐ strand, and a small domain with an α/β sandwich structure. In contrast, IDH3 forms a heterotetramer (α_2_βγ) from two heterodimers (αβ and αγ), comprising two α‐subunits, one β‐subunit, and one γ‐subunit. Like other IDH enzymes, it undergoes an “open‐to‐closed” conformational transition during catalysis. For IDH3, this transition—essential for catalytic activation—is induced by allosteric activators (e.g., citrate and ADP) and subsequent interdomain/inter‐subunit communication [56, 57].

Mutations in *IDH1/2* cause the enzyme to lose its normal catalytic ability (the oxidation of isocitrate to α‐KG) and gain a neomorphic function: the NADPH‐dependent reduction of α‐KG to D‐2HG [9]. This results in D‐2HG production that far exceeds the cell's metabolic clearance capacity, leading to significant accumulation within mutant tumors. Although trace amounts of D‐2HG exist in healthy organisms, its physiological role remains unknown [58]. Although the full mechanisms by which mIDH drives tumorigenesis are still being investigated, the downstream consequences of D‐2HG accumulation are well‐characterized. As an oncometabolite, D‐2HG is structurally similar to α‐KG and acts as a competitive inhibitor of α‐KG‐dependent dioxygenases [12], including histone lysine demethylases (KDMs) and the ten‐eleven translocation (TET) DNA demethylase family [13, 14]. mIDH induces epigenetic alterations leading to DNA and histone hypermethylation, a central mechanism driving tumorigenesis. Noushmehr et al.’s analysis of DNA methylation changes in GBM samples from the Cancer Genome Atlas (TCGA) database revealed a distinct glioma CpG island methylation phenotype, characterized by DNA methylation occurring predominantly at CpG islands [33, 42]. TET enzymes, which are α‐KG‐dependent, normally promote DNA demethylation by converting 5‐methylcytosine (5mC) to 5‐hydroxymethylcytosine (5hmC) [59]. By inhibiting TET enzymes, D‐2HG causes DNA hypermethylation [13]. Most of the DNA methylation sites altered by mIDH can gradually revert to their normal state after the mutant signal is abolished, whereas approximately 25% of the differentially methylated sites remain persistently abnormal, potentially influencing tumor development and recurrence [60]. Furthermore, D‑2HG promotes DNA hypermethylation by binding to DNMT1 and stimulating its association with the RIP3 gene promoter, inducing promoter hypermethylation and suppressing RIP3 expression [61]. Histone demethylases also play critical roles in cancer biology; similarly, D‐2HG competitively inhibits these enzymes, resulting in histone hypermethylation and impaired cellular differentiation [62]. The Jumonji C (JmjC) domain‐containing histone demethylase family catalyzes the demethylation of various histones, including H3K4, H3K9, H3K27, H3K36, and H4K20 [63]. High concentrations of D‐2HG inhibit KDM families such as KDM4 and KDM5 [64]. Understanding these precise mechanisms of epigenetic reprogramming is vital for developing targeted therapies.

mIDH also influences hypoxia‐inducible factors (HIFs) signaling via D‐2HG‐mediated inhibition of prolyl hydroxylases (PHDs), though the full regulatory consequences remain a subject of debate. HIFs are oxygen‐sensitive transcription factors (comprising HIF‐1α and HIF‐1β subunits) that regulate pathways critical for tumor growth [65], including angiogenesis and glucose metabolism [66]. Under normoxic conditions, PHDs hydroxylate HIF‐1α proline residues, marking them for ubiquitination and proteasomal degradation [67]. Reduced cellular α‐KG levels resulting from glioma‐derived IDH1 mutations impair the activity of PHDs, leading to HIF‐1α stabilization and the activation of target genes such as vascular endothelial growth factor (VEGF) [68], which can promote tumor proliferation and invasion. However, the impact of mIDH on HIF signaling appears context‐dependent; some studies report the downregulation of angiogenesis markers in mIDH gliomas, potentially due to tissue‐specific effects [23]. Additionally, D‐2HG can activate the HIF prolyl 4‐hydroxylases of the EGLN family (primarily EGLN1 and EGLN2), accelerating the hydroxylation and degradation of HIF‐1α, downregulating HIF activity, and thereby promoting tumorigenesis [69].

D‐2HG competitively inhibits the activity of branched‐chain amino acid transaminases 1 and 2 (BCAT1/2), leading to the accumulation of branched‐chain amino acids (BCAAs) and a decrease in the levels of branched‐chain α‐keto acids (BCKAs) and glutamate. To compensate for the defect in glutamate synthesis, IDH‐mutant cells upregulate the glutamine‐dependent glutaminase (GLS) pathway. The reduction in glutamate directly impairs the synthesis of glutathione (GSH), compromising cellular antioxidant capacity and rendering cells highly sensitive to oxidative stress, thereby creating a targetable metabolic vulnerability [70, 71].

Accumulation of the oncometabolite D‑2HG in IDH‐mutant tumors affects genome stability by targeting the ALKBH family of α‑ketoglutarate‐dependent dioxygenases. D‑2HG competitively inhibits ALKBH2 and ALKBH3, enzymes responsible for repairing alkylated DNA bases [72, 73]. This inhibition leads to DNA damage accumulation; elevated D‐2HG impairs DNA repair by inhibiting α‐KG‐dependent ALKBH enzymes, making mIDH cells more sensitive to alkylating agents [74]. In addition to direct enzymatic inhibition, D‑2HG can modulate DNA repair through epigenetic mechanisms. By interfering with α‑KG‐dependent dioxygenase activity, including ALKBH enzymes, it alters chromatin structure and represses DNA repair gene expression, further compromising genome integrity [33]. mIDH also induces epigenetic silencing of nicotinate phosphoribosyltransferase 1, lowering intracellular NAD^+^ levels and forcing cancer cells to depend on the NAD+ salvage pathway [75]. These dual effects, direct enzymatic inhibition and indirect epigenetic repression, underscore a multilayered regulatory axis through which D‑2HG modulates DNA repair and tumorigenesis. Functionally, this D‑2HG–ALKBH axis not only contributes to the enhanced mutation load observed in IDH‐mutant gliomas but also establishes potential vulnerabilities that can be exploited therapeutically. The impaired DNA repair capacity renders tumor cells more sensitive to DNA‐damaging agents, providing a rationale for synthetic lethal strategies targeting DNA repair deficiency. Particularly, the inhibition of the DNA repair enzymes of the ALKBH family is a key link connecting tumor metabolism and genomic instability. The deficiency of mismatch repair (MMR) in IDH‐mutant cancer cells leads to a hypermutated phenotype, potentially sensitizing these tumors to immune checkpoint inhibitors.

Furthermore, the interplay between metabolic reprogramming and epigenetic modulation highlights the complex crosstalk between oncometabolites and genome maintenance machinery, emphasizing the broader role of D‑2HG in shaping tumor evolution and therapeutic response [33]. In head and neck squamous cell carcinoma (HNSCC), programmed death‐ligand 1 (PD‐L1) undergoes nuclear translocation and directly binds genomic DNA, a mechanism identified through CRISPR synthetic lethal screening. This interaction regulates gene expression to reduce reactive oxygen species (ROS) and safeguard against ferroptosis. Consequently, strategies that block PD‐L1 nuclear entry may provide a synergistic approach to enhance immune checkpoint blockade (ICB) therapy [76]. Wang et al. found that ATR inhibitors induce synthetic lethality by promoting microsatellite DNA damage and trigger antitumor immunity via cGAS pathway activation, thereby enhancing programmed death 1 (PD‐1)‐targeted immunotherapy for MMR‐deficient cancers [77]. By deleting the lysine‐specific demethylase 1 gene and combining with the use of PD‐1 inhibitors, the synthetic lethal effect can effectively inhibit the growth and recurrence of HCC tumors and reshape TME, overcoming the drug resistance of advanced cancer patients [78]. Collectively, D‐2HG accumulation acts as both a pathogenic driver and a therapeutic vulnerability in mIDH malignancies, serving as a diagnostic biomarker and a target for intervention. By leveraging DNA repair defects through synthetic lethality, exploring the mechanisms underlying tumor cell death resulting from overactivated oncogenic pathways offers a highly promising approach to address the challenges of tumor heterogeneity and drug resistance.

### 2.2 mIDH and the Tumor Immune Microenvironment

The discovery that mIDH exerts profound immunosuppressive effects has expanded our understanding of their tumorigenic mechanisms beyond epigenetic alterations [79]. Beyond disrupting chromatin architecture and cellular differentiation programs, the oncometabolite D‐2HG acts as a potent immunomodulator that systematically impairs antitumor immunity across multiple effector mechanisms [80, 81]. This immune evasion phenotype contributes significantly to tumor progression and presents both challenges and opportunities for therapeutic intervention, particularly regarding the integration of IDH‐targeted therapies with immunomodulatory strategies [82].

#### 2.2.1 Direct Immunosuppressive Mechanisms of 2‐HG

The millimolar concentrations of D‐2HG that accumulate within IDH‐mutant tumor cells do not remain compartmentalized; instead, they diffuse into the extracellular space, where the metabolite directly encounters and impairs infiltrating immune cells [83]. Multiple complementary mechanisms converge to create a profoundly immunosuppressive microenvironment that undermines both innate and adaptive antitumor responses.

T lymphocytes are primary targets of D‐2HG‐mediated immunosuppression. Following uptake via the plasma membrane transporter SLC13A3, D‐2HG disrupts T‐cell activation by interfering with the calcium‐NFAT and NF‐κB signaling pathways, resulting in the dose‐dependent suppression of proliferation and effector function [27]. At concentrations of 20 mM, which are readily achieved in the IDH‐mutant TME, D‐2HG inhibits more than 50% of T‐cell proliferative capacity. The mechanistic basis for this suppression involves metabolic disruption: D‐2HG impairs mitochondrial ATP production, activating the AMPK energy‐stress pathway while simultaneously reducing ornithine decarboxylase activity and polyamine synthesis, processes essential for the biosynthetic demands of proliferating lymphocytes [27, 84]. Importantly, these inhibitory effects manifest in both CD4^+^ and CD8^+^ T‐cell subsets, with CD4^+^ helper T cells showing particular vulnerability, resulting in diminished production of critical cytokines such as interferon‐γ (IFN‐γ) and IL‐2.

The immunological consequences extend beyond functional impairment to affect T‐cell recruitment and phenotype. IDH‐mutant tumor cells exhibit suppressed secretion of CXCL10, a key chemokine involved in recruiting T cells to the tumor bed, mediated by D‐2HG‐driven inhibition of the STAT1 signaling pathway [84, 85]. This reduced chemokine production leads to the markedly lower tumor‐infiltrating lymphocyte (TIL) densities consistently observed in IDH‐mutant gliomas compared to their wild‐type (WT) counterparts. Notably, diminished NFAT signaling following D‐2HG exposure has also been associated with reduced PD‐1 expression on tumor‐infiltrating T cells; this may explain the limited efficacy of immune checkpoint inhibitors as monotherapy in IDH‐mutant malignancies, despite their success in other cancers [27, 86].

Natural killer (NK) cells experience similar suppression through distinct molecular mechanisms. The D‐2HG‐driven DNA hypermethylation signature extends to the genes encoding NKG2D ligands, including ULBP1 and ULBP3, on the tumor cell surface, substantially impairing NK‐cell recognition [87, 88]. The functional result is profoundly reduced NK‐cell‐mediated cytotoxicity and IFN‐γ production upon contact with IDH‐mutant targets. Critically, this epigenetic silencing mechanism appears reversible: treatment with the demethylating agent decitabine restored NKG2D ligand expression and resuscitated NK‐cell cytotoxic function. In murine models, this enhanced NK‐cell infiltration and provided significant tumor control benefits [87, 88].

#### 2.2.2 Impaired Antigen Presentation and Dendritic Cell (DC) Dysfunction

Professional antigen‐presenting cells within IDH‐mutant tumors exhibit profound functional deficits that compromise their ability to initiate adaptive immune responses. Although DCs infiltrating IDH‐mutant gliomas are often present at appreciable densities, they demonstrate an immature phenotype characterized by reduced expression of major histocompatibility complex (MHC) Class II molecules and the co‐stimulatory receptors essential for T‐cell activation [89]. This state of maturational arrest is largely attributable to the immunomodulatory effects of the oncometabolite D‐2HG. Experimental exposure of DCs to D‐2HG has been shown to suppress human leukocyte antigen (HLA)‐DR and IL‐6 expression while concomitantly inducing tolerogenic markers such as CD206 and PD‐L1, thereby skewing DCs toward an immunosuppressive rather than immunostimulatory functional state [89]. The functional consequences include impaired antigen presentation and a reduced ability to activate tumor‐specific T‐cell responses, creating a fundamental obstacle to effective antitumor immunity even when tumor neo‐antigens are abundant.

The broader myeloid compartment undergoes comparable immunomodulatory alterations in IDH‐mutant gliomas. Tumor‐associated macrophages (TAMs) and resident microglia exhibit altered polarization states and are present at reduced infiltration density, a phenomenon linked to diminished tumor‐derived expression of myeloid chemoattractants, including CCL2, CXCL2, and complement component C5 [90]. Single‐cell transcriptomic analyses have further revealed substantial heterogeneity within these myeloid populations; notably, IDH‐mutant tumors contain lower proportions of pro‐inflammatory and phagocytic macrophage subsets compared to WT tumors [80, 91]. This remodeling of the myeloid compartment extends to the complement system, as D‐2HG directly inhibits both classical and alternative complement pathway activation. This impairs C3b and C5b deposition, consequently reducing complement‐mediated cytolysis and opsonization [83].

#### 2.2.3 The Immune Checkpoint Paradox in IDH‐Mutant Tumors

The relationship between mIDH and immune checkpoint expression presents an apparent paradox with important therapeutic implications. IDH‐mutant tumors consistently demonstrate lower PD‐L1 expression than WT tumors, a result of increased PD‐L1 promoter methylation driven by D‐2HG‐mediated epigenetic reprogramming [92, 93]. Concurrently, tumor‐infiltrating T cells in IDH‐mutant contexts exhibit decreased PD‐1 expression due to impaired NFAT signaling [27]. As a result, both the ligand and the receptor components of the PD‐1/PD‐L1 immune checkpoint axis are attenuated within the TME. This molecular configuration, combined with the general paucity of TILs, renders IDH‐mutant tumors largely “immune cold” and poorly responsive to PD‐1/PD‐L1 checkpoint blockade as a monotherapy.

However, recent high‐resolution immune profiling has revealed substantial heterogeneity within IDH‐mutant tumors. Single‐cell analyses have identified subsets of IDH‐mutant gliomas, particularly those with high protein‐level immune microenvironment scores, that harbor significant populations of exhausted T cells expressing multiple checkpoint molecules, including PD‐1, T‐cell immunoglobulin mucin 3 (TIM‐3), and LAG3 [94]. Collectively, these findings demonstrate continuous variability in immune checkpoint expression and T cell exhaustion among IDH‐mutant malignancies, a critical premise for identifying candidates responsive to checkpoint inhibitors. The transcription factor aryl hydrocarbon receptor has emerged as a key regulator of T‐cell exhaustion in these contexts, representing a potential target for reversing immune suppression [91].

#### 2.2.4 Therapeutic Implications and Combination Strategies

Comprehensive characterization of D‐2HG‐mediated immunosuppression has provided a mechanistic framework for rational therapeutic strategies that target both the metabolic driver and its downstream immune consequences. IDH inhibitors function not only as antiproliferative agents by restoring cellular differentiation but also as immunomodulators by reducing D‐2HG levels and alleviating immune suppression. These observations support the rationale for combining IDH inhibitors with immunotherapeutic strategies to overcome tumor‐associated immunosuppression [84, 85].

Vaccination strategies targeting the IDH1 R132H neoantigen have shown promising preclinical efficacy, induced robust Th1‐polarized immune responses, and generated mutation‐specific antibodies capable of recognizing IDH‐mutant cells [95]. Early clinical studies suggest IDH1‐targeted vaccines are well‐tolerated and capable of eliciting immune responses, though clinical efficacy data remain preliminary. Combining IDH vaccination with IDH inhibitors is a promising approach, as metabolic normalization may enhance the TME's receptivity to vaccine‐elicited immune responses.

Epigenetic modulation via demethylating agents is another validated strategy, particularly for reversing NK‐cell‐mediated immune evasion. Decitabine treatment successfully unmasks NKG2D ligands silenced by D‐2HG‐driven hypermethylation, restoring tumor cell recognition by NK cells [87, 88]. The selective dependence of this effect on NK‐cell function, confirmed through depletion experiments, establishes a clear mechanistic basis for NK‐cell‐directed combination therapies.

Novel immunomodulatory strategies specifically tailored to IDH‐mutant tumors are also emerging. Nanoparticle‐based “immuno‐initiator” platforms, capable of inducing immunogenic cell death, restoring T‐cell populations, and synergizing with PD‐1 blockade, have shown preclinical efficacy in IDH‐mutant glioma models [96]. Additionally, the metabolic engineering of adoptively transferred T cells using IDH2 inhibitors to reprogram their epigenetic and metabolic states has demonstrated enhanced memory phenotype formation and superior antitumor efficacy in hostile microenvironments [97]. These approaches exemplify the translation of mechanistic insights into rationally designed immunotherapies tailored to the unique biology of IDH‐mutant malignancies.

#### 2.2.5 Clinical Context and Future Directions

The profound influence of mIDH status on tumor immune architecture necessitates its consideration in clinical decision‐making related to immunotherapy eligibility and trial design. Despite a generally favorable prognosis due to preserved differentiation and slower growth, IDH‐mutant tumors present formidable immunological challenges due to their “cold” phenotype. Conversely, specific IDH‐WT subsets, particularly those with DNA MMR deficiency and resultant hypermutation, demonstrate “hot” characteristics with substantial lymphocyte infiltration and superior responses to checkpoint inhibition [98]. Accordingly, molecular stratification incorporating IDH status, mutational burden, and immune profiling is increasingly used to guide immunotherapy selection.

Emerging spatial omics technologies—including Visium, CosMx, Xenium in situ sequencing, GeoMx Digital Spatial Profiling, and multimodal platforms such as CODEX spatial proteomics—have enabled spatially resolved investigation of how mIDH shape tumor heterogeneity. In high‐grade gliomas (HGG), integrative analyses across multiple spatial platforms indicate that the IDH genotype is a key determinant of the spatial architecture of tumor heterogeneity. IDH‐WT glioblastoma exhibits substantially greater heterogeneity, characterized by mesenchymal transcriptional programs within tumors and glial lineage divergence between tumors. In contrast, IDH‐mutant gliomas display relatively limited heterogeneity, with tumor cells largely restricted to an oligodendroglial lineage state, whereas progenitor‐like populations are primarily observed in proliferative regions. These spatial organizational differences provide a mechanistic basis for the distinct prognostic outcomes associated with the two molecular subtypes [99, 100]. Recent spatial omics studies have provided new insights into the ecological organization of gliomas. Two core spatial niches have been identified: an intracerebral niche composed primarily of glioma cells and microglia and an extracerebral niche enriched with mesenchymal cells and monocytes/macrophages, establishing a spatial framework that supports the development of niche‐targeted therapeutic strategies. Further spatial transcriptomic analysis using Visium on formalin‐fixed paraffin‐embedded samples revealed that IDH‐mutant tumor regions are enriched in MAPK and JAK–STAT signaling pathways and exhibit a higher degree of immune cell infiltration compared with IDH‐WT tumors. These findings indicate that IDH‐mutant gliomas possess a distinct spatial immune infiltration landscape and have enabled the identification of potential therapeutic targets, including SPP1 [101]. Building on these observations, spatiotemporal multi‐omics profiling of IDH‐mutant astrocytomas further stratified tumors into four proteomic subtypes—adipogenesis/fatty‐acid metabolism, proliferative/progenitor, immune/mesenchymal‐enriched (IME), and neuronal. Among them, the IME subtype is associated with the poorest prognosis and displays a layered spatial architecture characterized by perivascular lymphocytic cuffs. Notably, this subtype shows a paradoxical immune phenotype combining an immune‐inflamed microenvironment with pronounced T‐cell exhaustion and enrichment of glioblastoma‐like differentiation signatures, thereby refining the molecular classification of IDH‐mutant astrocytomas and underscoring their translational relevance [101, 102].

At the level of TME regulation and disease progression, the integrated application of spatial omics and multi‐omics approaches has provided systematic insights into the mechanisms driving recurrence of IDH‐mutant gliomas. Studies of recurrent IDH‐mutant tumors have shown that both the mTORC1 signaling pathway and angiogenic programs are coordinately activated at recurrence. AURKA has been identified as a key upstream kinase responsible for driving mTORC1 activation. In addition, a previously unrecognized subset of TAMs, termed angio‐TAMs, was characterized as a pro‐tumorigenic population. Spatial analyses further demonstrated that the AURKA–mTORC1 signaling axis promotes the polarization of Angio‐TAMs, thereby cooperatively driving tumor recurrence and angiogenesis in IDH‐mutant gliomas. These findings provide important evidence supporting targeted therapeutic strategies for recurrent disease [103]. In terms of immune regulation, recent studies have shown that IDH‐mutant tumors suppress the expression of chemokine and cytokine genes such as CCL5 and IL‐6 through a hypermethylation‐dependent mechanism, thereby fundamentally limiting the recruitment of myeloid‐derived suppressor cells (MDSCs). In contrast, IDH‐WT tumors harbor two distinct MDSC subsets—early‐stage MDSCs (E‐MDSCs) and monocytic MDSCs (M‐MDSCs)—which form a barrier‐like spatial arrangement with stem‐like tumor cells, establishing a highly immunosuppressive microenvironment that supports tumor progression [104].

This epigenetic mechanism of immune regulation differs fundamentally from that observed in IDH‐WT tumors. An additional integrative study combining single‐cell RNA sequencing and spatial transcriptomics demonstrated that, in IDH‐WT gliomas, IRF7‐mediated interferon signaling is markedly overactivated and actively orchestrates the recruitment and functional programming of myeloid cells, thereby promoting the establishment of a tumor‐promoting microenvironment [105]. These results highlight a fundamental difference in TME regulation between IDH genotypes, whereby IDH‐mutant tumors evade immune surveillance via epigenetic immune silencing, while IDH‐WT tumors drive progression through active myeloid cell recruitment.

Taken together, the integration of spatial transcriptomics with multi‐omics approaches is progressively reshaping the conceptual framework of IDH‐mutant glioma biology, spanning spatial heterogeneity, molecular classification, microenvironmental regulation, and mechanisms of recurrence. These advances provide an important basis for the development of precision‐targeted therapies and rational combinatorial immunotherapeutic strategies.

## 3 Bonding Bench to Bedside: Relevant Preclinical Advances

Since the initial discovery in GBM in 2008, IDH1 and IDH2 mutations have been identified in over 20 tumors, establishing their status as therapeutically actionable oncogenic drivers across diverse malignancies. The translation from mechanistic research to clinical applications has been enabled by rigorous preclinical studies employing genetically engineered cell lines, patient‐derived xenograft (PDX) models, blood–brain barrier (BBB)‐penetrant compounds, and three‐dimensional (3D) culture systems. These models have not only validated the pathogenic role of mutant IDH‐driven oncometabolism but have also guided the rational design of FDA‐approved inhibitors, informed combination therapy strategies, and elucidated resistance mechanisms.

### 3.1 Prevalence of mIDH in Cancers

**FIGURE 2:**
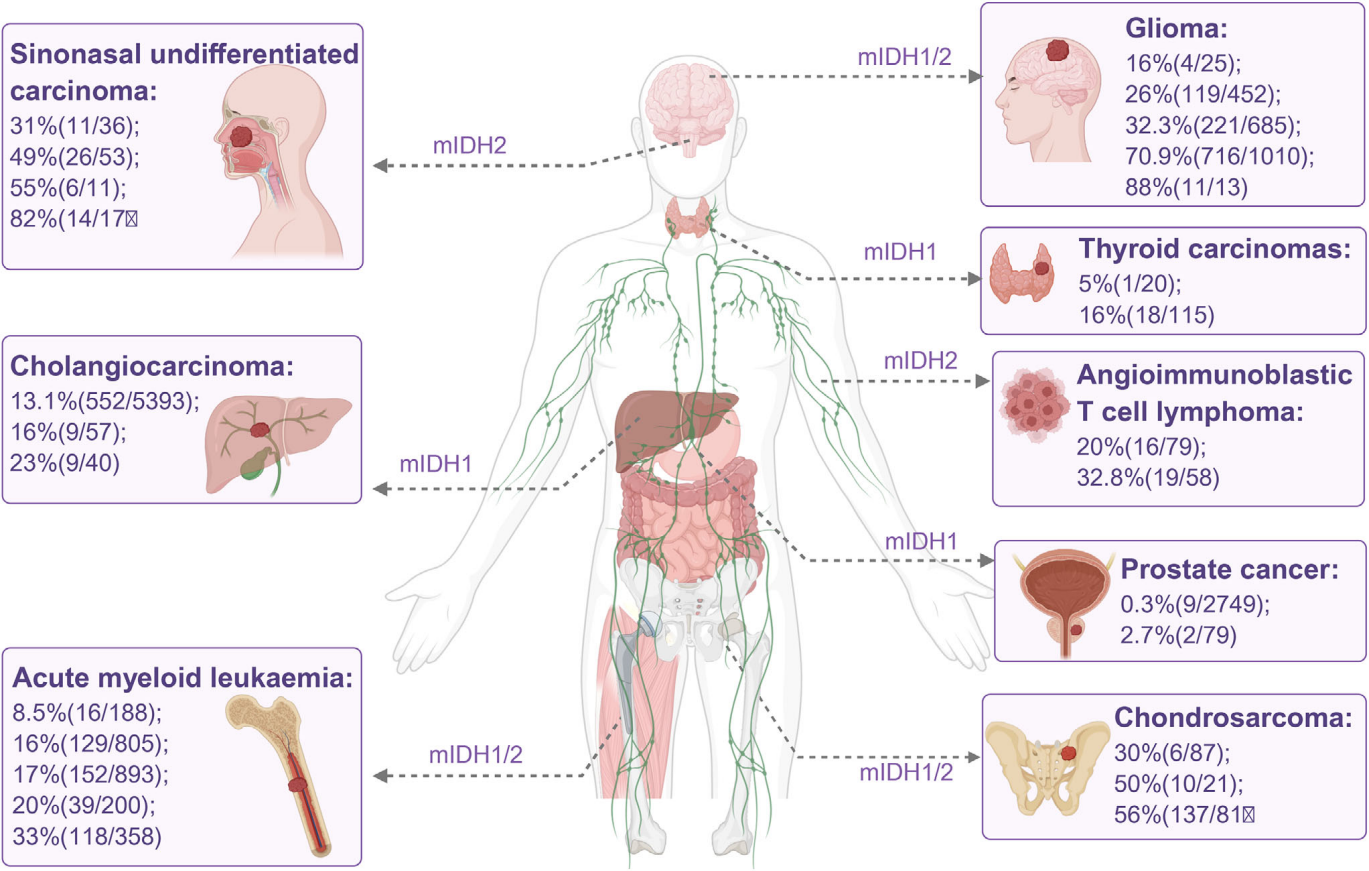
Frequency and distribution of IDH mutations in different tumor types. IDH1 and IDH2 are mutated at varying rates in a range of cancers, mainly low‐grade gliomas (LGGs) and secondary glioblastomas, acute myeloid leukemia (AML), cholangiocarcinoma (CCA), central and periosteal chondrosarcoma, sinonasal undifferentiated carcinoma (SNUC), angioimmunoblastic T‐cell lymphoma (AITL), thyroid carcinomas, and prostate cancers. The frequency and types of IDH mutations in each cancer type are shown. IDH, isocitrate dehydrogenase; mIDH, mutant isocitrate dehydrogenase.

mIDH was first discovered in human tumors in 2008 when Parsons et al. sequenced the genomes of 22 patients with GBM, revealing that 12% of these patients harbored IDH1 mutations at position R132 [1]. Since then, mIDH have been identified in a wide variety of tumor types (Figure 2). To date, IDH1 and IDH2 mutations have been found in more than 20 tumor types [47], with the highest frequencies observed in gliomas (16%–88%) [41, 46, 106, 107, 108], AML (16%–20%) [2, 3, 4], CCA (13.1%–23%) [5, 44, 109], central and periosteal chondrosarcoma (30%–56%) [6, 7, 45], sinonasal undifferentiated carcinoma (SNUC, 31%–82%) [48, 49, 50, 110, 111, 112], and angioimmunoblastic T‐cell lymphoma (AITL, 20%–32.8%) [51, 52, 113, 114, 115]. These mutations have also been reported in other cancers, including thyroid carcinomas (5%–16%) and prostate cancers (0.3%–2.7%) [46, 53, 54, 116].

mIDH is typically heterozygous and cluster at specific hotspot residues. Mutations in IDH1 predominantly affect the arginine residue at position 132, whereas IDH2 mutations are concentrated at positions R140 and R172. Notably, IDH1 and IDH2 mutations are almost mutually exclusive [117]. In AML, the frequency of mIDH is highly age‐dependent, increasing from 3.4% in pediatric patients to 21% in those over 60 years old [43]. Furthermore, mIDH status significantly influences clinical outcomes and prognosis [118]. In AML patients, IDH1 mutations are associated with poorer overall survival (OS; HR 1.17, 95% CI: 1.05–1.31, *p* = 0.0047), whereas IDH2 mutations confer a better prognosis (HR 0.79, 95% CI: 0.66–0.93) and a lower risk of death [119, 120]. Among IDH2‐mutant AML cases, the R140 mutation is associated with significantly improved OS and prognosis compared to the R172 mutation, particularly in younger patients [121].

### 3.2 Preclinical Models of mIDH

**FIGURE 3:**
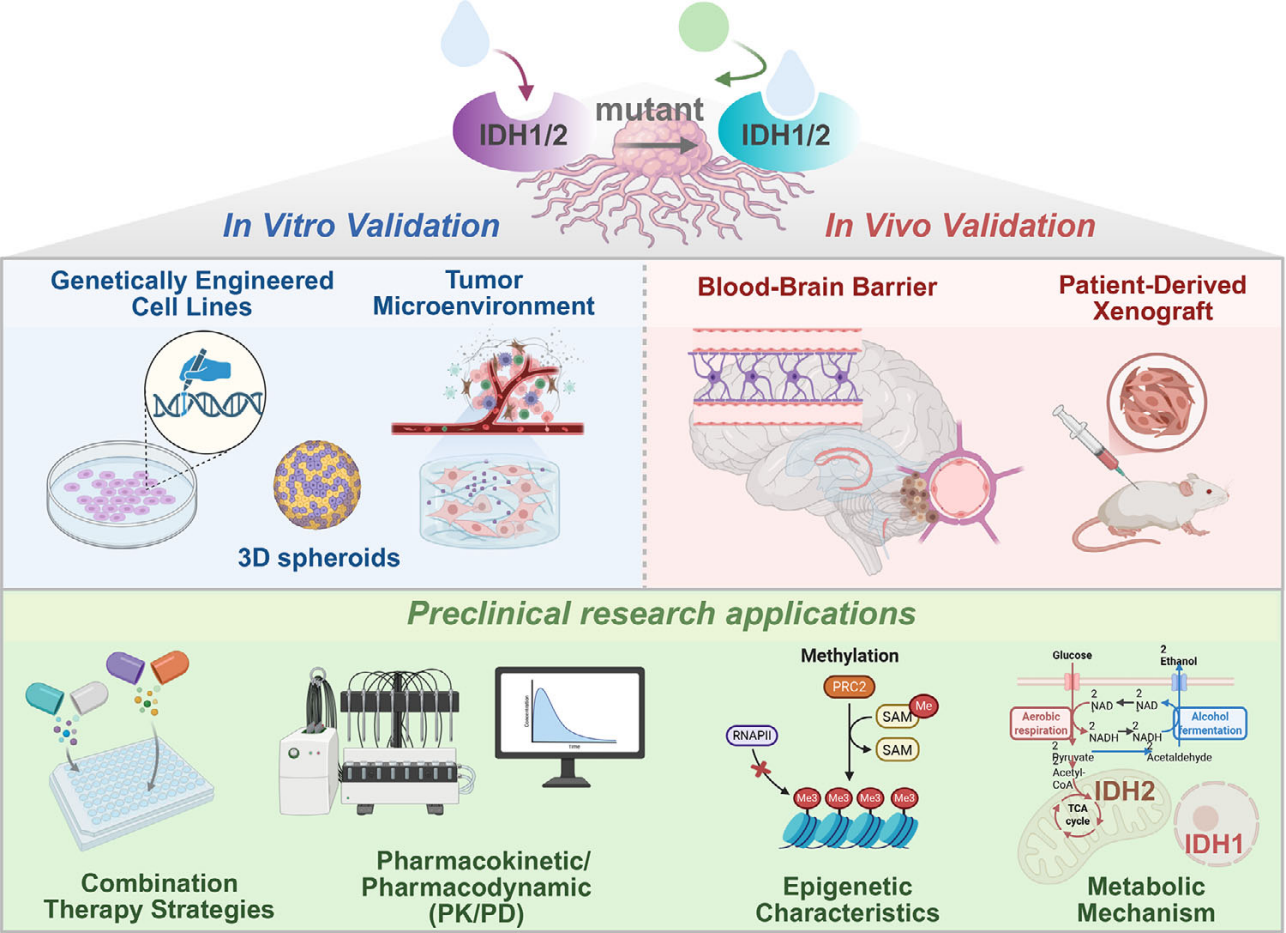
Preclinical research applications targeting mutant IDH using various experimental models. In vivo and in vitro experimental models recapitulating key features of human mIDH‐driven cancers (e.g., tissue‐specific mutation patterns, D‐2HG accumulation, epigenetic signatures, and TME interactions) confirmed the pathogenic role of mIDH across multiple cancer types, validating the efficacy of combinatorial regimens and uncovering the molecular and metabolic mechanisms underlying treatment response and resistance. IDH, isocitrate dehydrogenase.

Preclinical investigations on mIDH have utilized a diverse array of experimental models, including genetically engineered cell lines, PDXs, orthotopic tumor models, and metabolically stratified systems, to address critical questions in translational oncology. These models have confirmed the pathogenic role of mIDH across multiple cancer types, including AML, glioma, chondrosarcoma, and CCA. Moreover, preclinical mIDH models have served as essential tools for optimizing the development of mIDH‐targeted therapies, validating the efficacy of monotherapies and combinatorial regimens, and uncovering the molecular and metabolic mechanisms underlying treatment response and resistance. By recapitulating key features of human mIDH‐driven cancers, such as tissue‐specific mutation patterns, D‐2HG accumulation, epigenetic signatures, and TME interactions, preclinical models have bridged the gap between basic mechanistic insights and clinical application [122]. These models have guided the development of FDA‐approved inhibitors (e.g., ivosidenib, enasidenib, and vorasidenib) and informed the design of next‐generation therapeutic strategies (Figure 3).

#### 3.2.1 Cell‐Based Models and In Vitro Validation

Cell‐based systems constitute the primary platform for initial drug screening and mechanistic interrogation of mIDH inhibitors. Ivosidenib (AG‐120), the first FDA‐approved mIDH1 inhibitor, demonstrated potent and selective activity across multiple IDH1‐R132 mutant subtypes (R132H, R132C, and R132G) in both endogenous and engineered cell lines. In HT1080 chondrosarcoma cells harboring the IDH1‐R132C mutation, ivosidenib exhibited a cellular IC_50_ of 8 nM, whereas U87 MG glioblastoma cells (IDH1‐R132H) showed an IC_50_ of 19 nM, with enzymatic IC_50_ values consistently ranging between 8 and 13 nM [123]. Critically, ivosidenib reduced intracellular D‐2HG levels by 96% at 0.5 µM in primary AML patient cells. This reduction concomitantly induced myeloid differentiation, as evidenced by enhanced colony‐forming capacity, upregulation of surface differentiation markers (CD14 and CD15), and increased proportions of mature myeloid cells, effects that were entirely absent in IDH1‐WT cells [123].

Similarly, BAY1436032 demonstrated exquisite IDH1 mutation specificity in primary human AML cells, with D‐2HG inhibition IC_50_ values of 3–16 nM across various IDH1‐R132 variants (H/C/G/L/S), while showing no inhibitory activity against IDH2‐mutant or IDH‐WT cells even at concentrations up to 10 µM []. At 0.1 µM, BAY1436032 suppressed colony formation by 50% in mIDH1 AML cells and reversed aberrant histone hypermethylation (H3K4me3 and H3K9me3). Mechanistically, these effects were linked to the de‐repression of the PU.1 promoter and the methylation‐mediated silencing of E2F1, thereby activating differentiation‐associated transcription factors while concomitantly suppressing cell‐cycle progression pathways [124].

The mIDH2 inhibitor enasidenib displayed mutation‐specific efficacy in chondrosarcoma models, with 72‐h cellular viability IC_50_ values of 16.7–22.7 µM for IDH2‐R172G (CDS17 cells) and 49.5 µM for IDH2‐R172S (SW1353 cells), contrasting with an IC_50_ > 100 µM in IDH‐WT CDS23 cells [125]. Transcriptomic analyses revealed that enasidenib‐mediated growth inhibition occurred independently of global DNA methylation changes or histone modification patterns (H3K4me3, H3K9me3, and H3K27me3). Its anti‐tumor activity was associated with modulation of TGF‐β, mTORC1, and E2F‐mediated signaling, as well as G2/M checkpoint pathways [125].

#### 3.2.2 BBB Penetration and Central Nervous System (CNS)‐Active Compounds

A critical limitation of first‐generation mIDH inhibitors (ivosidenib and enasidenib) has been inadequate BBB penetration, which constrains their efficacy against IDH‐mutant gliomas. DS‐1001b, a novel orally bioavailable mIDH1 inhibitor, was specifically engineered to overcome this barrier. Structural studies revealed that DS‐1001b binds to an allosteric pocket on the IDH1‐R132C homodimer surface, stabilizing an “open” inactive conformation by disrupting the catalytic aspartate residue's spatial arrangement and reducing α‐KG binding affinity. In cell‐based assays, DS‐1001b exhibited IC_50_ values of ∼30 nM for IDH1‐R132H and ∼200 nM for other R132 variants (C/G/L/S) in D‐2HG production assays [126, 127].

Pharmacokinetic evaluations demonstrated that oral administration of [^14^C]DS‐1001a in mice yielded brain‐to‐plasma profiles with consistent temporal kinetics, achieving a brain area under curve (AUC) equivalent to 65% of the plasma (*r* = 0.997, *p* < 0.001). In subcutaneous A1074 PDX models (IDH1‐R132H), 4 weeks of continuous DS‐1001b treatment significantly suppressed tumor growth (*p* < 0.001), reduced intratumoral D‐2HG levels (*p* = 0.003), and induced expression of the astrocytic differentiation marker GFAP, with no effect on IDH‐WT tumors [126]. Notably, 10 weeks of DS‐1001b therapy resulted in marked tumor reduction confirmed by MRI and histological analyses (*p* = 0.006), sustained D‐2HG suppression, and GFAP upregulation, with no observable toxicity [126].

Vorasidenib (AG‐881), a dual mIDH1/2 inhibitor, represents a paradigm shift in addressing therapeutic coverage and BBB permeability. Optimized from the enasidenib analog AGI‐12026, vorasidenib was designed to fulfill key CNS penetration criteria: a hydrogen bond donor count ≤2, a topological polar surface area (tPSA) of 73–86Å² , and log*P* > 3 [127]. X‐ray structures demonstrate that vorasidenib binds symmetrically to the allosteric pockets of both mIDH1‐R132H and mIDH2‐R140Q enzymes, engaging conserved residues (Q277/Q316, D273/D312) through hydrogen bonding and halogen interactions. Meanwhile, the CF_3_ moiety establishes van der Waals contacts with hydrophobic residues (V255, M259), stabilizing the open inactive conformation. Biochemical IC_50_ values were remarkably low: 0.6 nM for mIDH1‐R132H heterodimers and 7 nM for mIDH2‐R140Q [127].

In pharmacokinetic studies, vorasidenib achieved brain‐to‐plasma ratios of 0.65–1.33 across preclinical species following single oral doses of 3 mg/kg, with brain and cerebrospinal fluid (CSF) concentrations tracking plasma levels proportionally. In TS603 IDH1‐R132H orthotopic glioma models, 50 mg/kg twice‐daily dosing for 4 days suppressed brain tumor 2‐HG production by >97%, with a brain tumor‐to‐plasma AUC ratio of 1.25 and sustained inhibition exceeding 24 h [127]. Early clinical data have confirmed that vorasidenib reduces 2‐HG levels by >90% in human mIDH gliomas, validating its translational potential for low‐grade glioma (LGG) therapy.

#### 3.2.3 PDX Models: Bridging Preclinical and Clinical Paradigms

Xenograft models, including both cell line‐derived xenografts (CDX) and PDX, have emerged as indispensable tools for validating therapeutic efficacy and predicting clinical responses. In preclinical studies of ivosidenib, single oral doses of 50 or 150 mg/kg in BALB/c nude mice bearing HT1080 xenografts achieved maximal tumor D‐2HG suppression of 92.0% and 95.2% at 12 h post‐administration, respectively. Levels returned to baseline within 48–72 h, consistent with a reversible inhibition mechanism. Notably, ivosidenib demonstrated favorable pharmacokinetics across multiple species (rats, dogs, and cynomolgus monkeys), characterized by rapid oral absorption, low clearance, and a moderate half‐life. In rats, a single 50 mg/kg oral dose yielded a BBB penetration rate of 4.1%, suggesting potential therapeutic utility for gliomas [123].

BAY1436032 exhibited exceptional efficacy in two molecularly distinct AML PDX models. In PDX1 (IDH1‐R132C + FLT3‐TKD + NPM1 + NRAS), daily oral administration of 150 mg/kg maintained serum D‐2HG below 1 µM for 150 days, reduced peripheral blood hCD45^+^ cell frequency to 2.8% by Day 150, and achieved 100% survival to the study endpoint (compared to a median survival of 91 days in vehicle controls). In the late‐stage PDX2 model (IDH1‐R132C + MLL‐PTD), the same dose enabled 60% of mice to survive beyond 100 days, with a sustained low tumor burden persisting after treatment cessation [124].

Mechanistically, BAY1436032 induced in vivo AML cell differentiation, marked by decreased CD34^+^ stem cell markers and increased CD14^+^/CD15^+^ markers, and demonstrated profound LSC eradication, reducing LSC frequency 100‐fold compared to vehicle controls [124]. Similarly, IDH305 demonstrated dose‐dependent D‐2HG suppression in HCT116‐IDH1‐R132H/+ xenografts, where a single 200 mg/kg oral dose achieved 87.2% maximal inhibition and an estimated unbound EC_50_ of 0.02–0.03 µM, which is concordant with in vitro IC_50_ values. In HMEX2838 PDX models (IDH1‐R132C), single 300 mg/kg doses produced >98% D‐2HG suppression lasting 16–20 h. Furthermore, 21 days of twice‐daily dosing at 300 mg/kg maintained 97%–99% inhibition and induced a 32% partial tumor regression (*p* < 0.05) without body weight loss or overt toxicity [128].

#### 3.2.4 Combination Therapy Strategies: Synergistic Mechanisms and LSC Eradication

In vivo validation of combination strategies utilized two molecularly characterized AML PDX models in NOD‐SCID gamma (NSG) mice: AML‐PDX1 (IDH1‐R132C + FLT3‐TKD + NPM1 + NRAS) and AML‐PDX2 (IDH1‐R132H + DNA methyltransferase 3 alpha [DNMT3A] + protein tyrosine phosphatase non‐receptor Type 11 [PTPN11] + NPM1). Treatment arms included a vehicle, monotherapy (AZA or BAY1436032), sequential combination (AZA initiated on Day 1, followed by BAY1436032 on Day 6), and simultaneous combination (both agents initiated on Day 1). In the PDX1 model, simultaneous combination therapy achieved superior control of leukemic burden: 33% of mice remained leukemia‐free at 36 weeks, the median mIDH1 allelic burden was reduced to 10.89% (vs. 48.8% in controls), and 5/6 mice survived to the 300‐day endpoint. This significantly outperformed sequential combination (median survival 299 days) and monotherapy (AZA: 188 days; BAY1436032: 220 days) [129]. The PDX2 model corroborated these findings, with simultaneous combination sustaining low peripheral blood leukemia cell frequencies and achieving a median survival of 250 days. Limiting dilution transplantation assays demonstrated that simultaneous combination therapy reduced LSC frequency 33,150‐fold relative to the vehicle, representing 282‐fold and 70‐fold greater LSC depletion compared to monotherapy and sequential combination, respectively [129].

Mechanistic investigations revealed that simultaneous combination therapy potently suppressed two critical signaling axes: (1) the MAPK/ERK pathway (reduced p‐ERK1/2, ELK1, and Cyclin D1 expression) and (2) the Rb/E2F pathway (inhibited Rb phosphorylation at S795/S807/S811, blocking the G1/S phase transition) while concurrently upregulating myeloid differentiation markers (CD14 and CD15). Notably, MEK1/2 inhibitors (trametinib) and CDK4/6 inhibitors (abemaciclib) exhibited significantly greater inhibitory activity against mIDH1 versus WT AML cells, further validating these pathways as mutation‐specific therapeutic vulnerabilities [129]. These findings provide a critical preclinical rationale for the simultaneous, rather than sequential, initiation of mIDH inhibitor and AZA combination therapy in clinical practice.

#### 3.2.5 3D Culture Systems and TME Modeling

Advanced 3D culture platforms have proven invaluable for recapitulating TME features that modulate drug response. In chondrosarcoma models, DS‐1001b retained antiproliferative activity against IDH1‐mutant cells (JJ012, L835) cultured as 3D spheroids or under hypoxic conditions (1% O_2_), albeit with modestly elevated GI_50_ values (e.g., JJ012 spheroid GI_50_ = 1444 nM vs. 81 nM in 2D monolayers) and without inducing significant apoptosis [130]. Notably, distinct mechanistic responses emerged across chondrosarcoma subtypes. In conventional chondrosarcoma L835 cells, DS‐1001b reduced H3K9me3 levels at the SOX9 locus, leading to upregulation of key chondrocyte differentiation regulators (e.g., SOX9 and RUNX2) and downstream extracellular matrix genes (e.g., COL2A1, COL10A1, and ACAN), thereby promoting terminal chondrocytic differentiation and proteoglycan‐rich matrix deposition. Conversely, in dedifferentiated chondrosarcoma JJ012 cells, DS‐1001b decreased H3K9me3 at the CDKN1C locus, inducing expression of the cyclin‐dependent kinase inhibitor p57 (CDKN1C) and triggering G_0_/G_1_ arrest [130]. Overexpression of CDKN1C phenocopied the effects of DS‐1001b, while CDKN1C knockout abrogated drug sensitivity, confirming that p57‐mediated cell cycle arrest is the mechanistic basis for responsiveness in the dedifferentiated subtype.

#### 3.2.6 Pharmacokinetic/Pharmacodynamic (PK/PD) Relationships and Target Engagement

Establishing robust PK/PD relationships has been critical for dose selection and clinical trial design. BAY1436032 exhibited linear plasma exposure across the 45–150 mg/kg dose range, with unbound drug concentrations exceeding in vitro IC_50_ values for 24 h. Once‐daily oral administration at 150 mg/kg achieved near‐complete D‐2HG suppression, supporting this dose for efficacy studies [124]. IDH305 demonstrated highly consistent unbound plasma EC_50_ values (0.02–0.03 µM) across xenograft models, aligning with cellular IC_50_ measurements and validating target engagement biomarkers [128]. In HMEX2700 PDX models (IDH1‐R132G), single doses of 100 and 300 mg/kg IDH305 achieved 92% and 97% maximal D‐2HG suppression, respectively, establishing dose‐response relationships suitable for clinical extrapolation.

For vorasidenib, human PK predictions indicated that 150 mg twice daily or 500 mg once daily in a 70 kg adult would maintain unbound concentrations above in vitro IC_50_ thresholds. Phase I clinical data validated these projections; 150 mg single doses yielded a geometric mean *C*_max_ of 1.2 µM and an AUC of 6.6 µM h. These results deviated by less than 1.5‐fold from predicted values, underscoring the translational fidelity of preclinical PK/PD models [127].

#### 3.2.7 Resistance Mechanisms and Therapeutic Escape Pathways

Longitudinal preclinical studies have illuminated mechanisms underlying therapeutic resistance to mIDH inhibitors. In ivosidenib plus AZA combination therapy models, treatment‐emergent resistance was not attributable to IDH isoform switching or secondary IDH1 mutations, both of which are established mechanisms of ivosidenib monotherapy resistance. Instead, 75% of relapsed cases (9/12 patients) exhibited clonal evolution characterized by the acquisition of transcription factor mutations (e.g., RUNX1 and ETV6) or signaling pathway alterations (e.g., NRAS). Single‐cell sequencing revealed the clearance of IDH1^+^/NPM1^+^/NRAS^+^ clones and the selective expansion of IDH1‐negative subclone [29]. Cytometry by time‐of‐flight (CyTOF) analyses identified elevated expression of the anti‐apoptotic protein myeloid cell leukemia‐1 (MCL‐1) in residual cells from relapsed patients, contrasting with the effective elimination of BCL‐2‐dependent cell populations in responders. This implicates MCL‐1 upregulation as a potential escape mechanism [29]. Notably, patients harboring RAS/RTK pathway mutations derived greater benefit from triple combination therapy (ivosidenib + venetoclax + AZA), achieving 12‐month OS rates of 90% versus 38% with ivosidenib plus venetoclax alone. This highlights the therapeutic relevance of co‐targeting survival signaling in high‐risk molecular subgroups [29].

#### 3.2.8 Limitations and Translational Considerations

Despite their translational value, preclinical models possess inherent limitations that constrain clinical predictability. Immunodeficient xenograft systems (NSG, BALB/c nude mice) fail to recapitulate human immune system dynamics, precluding the assessment of immunomodulatory effects and immune‐mediated toxicities. Long‐term safety evaluations, including cardiotoxicity, hepatotoxicity, and secondary malignancy risks, remain underexplored in current preclinical paradigms. Furthermore, most PDX studies have excluded patients with complex co‐mutational backgrounds (e.g., FLT3‐ITD and TP53), limiting generalizability to the molecular heterogeneous populations encountered in clinical practice. The discordance between 2D and 3D culture systems, exemplified by the altered GI_50_ values for DS‐1001b in spheroid models, underscores the importance of the microenvironmental context in drug response. This necessitates the integration of patient‐derived organoids and humanized mouse models into next‐generation preclinical platforms. Table 2 provides a comprehensive summary of the preclinical model systems used to evaluate IDH‐targeted therapies, delineating their respective applications, key findings, methodological advantages, and inherent limitations.

**TABLE 2:** Preclinical models for evaluating isocitrate dehydrogenase (IDH)‐targeted therapies.

Model type	Representative system	IDH inhibitor(s) tested	Key findings	Advantages	Limitations	Reference
Cell‐based in vitro models	HT1080 (IDH1‐R132C), U87 MG (IDH1‐R132H), primary AML cells, chondrosarcoma cell lines	Ivosidenib, BAY1436032, enasidenib	Ivosidenib IC_50_: 8–19 nM (cellular), 8–13 nM (enzymatic); 96% D‐2HG reduction at 0.5 µM with myeloid differentiation induction. BAY1436032 IC_50_: 3–16 nM across IDH1 variants; reverses histone hypermethylation. Enasidenib IC_50_: 16.65–49.47 µM (IDH2‐mutant), >100 µM (WT)	High‐throughput drug screening; mechanistic interrogation of D‐2HG suppression and differentiation; rapid IC_50_ determination; cost‐effective	Lacks tumor microenvironment complexity; no immune cell interactions; limited in vivo PK/PD correlation	[123, 124, 125]
BBB penetration models	Mouse orthotopic glioma PDX (TS603, A1074, HT1080)	DS‐1001b, Vorasidenib	DS‐1001b: brain AUC 65% of plasma, IC_50_ ∼30 nM (IDH1‐R132H); 4–10 weeks treatment suppressed tumor growth (*p* < 0.001–0.006) and induced GFAP expression. Vorasidenib: biochemical IC_50_ 0.6 nM (mIDH1), 7 nM (mIDH2); brain:plasma ratio 0.65–1.33; >97% brain tumor D‐2HG suppression	Critical for CNS‐active drug assessment; evaluates brain distribution and glioma‐specific efficacy; validates clinical translation for brain tumors	Species differences in BBB permeability; extrapolation to human PK requires caution; immunodeficient hosts	[126, 127]
Patient‐derived xenografts (PDX)	AML PDX in NSG mice, chondrosarcoma HT1080 PDX in BALB/c nude mice	Ivosidenib, BAY1436032, IDH305	Ivosidenib 50–150 mg/kg: 92%–95% tumor D‐2HG suppression at 12 h. BAY1436032 150 mg/kg: 100% survival at 150 days, 33,150‐fold LSC frequency reduction, and maintained 2‐HG <1 µM. IDH305 300 mg/kg: >98% D‐2HG suppression, 32% partial regression	Preserves patient tumor heterogeneity and microenvironment architecture; predicts clinical efficacy and response; enables resistance mechanism investigation	Immunodeficient mice preclude immune response assessment, long‐term toxicity underexplored, and most studies exclude complex co‐mutational backgrounds	[123, 124, 128]
3D culture and hypoxia models	JJ012, L835 spheroids (IDH1‐mutant chondrosarcoma); 1% O_2_ hypoxic conditions	DS‐1001b	3D spheroid GI_50_: 1444 nM (JJ012), compared to 2D GI_50_: 81 nM (18‐fold increase). Induced differentiation markers (SOX9, RUNX2) in conventional chondrosarcoma and cell cycle inhibitor p57/CDKN1C in dedifferentiated cells	Recapitulates 3D matrix architecture and hypoxic tumor microenvironment; evaluates drug penetration and spatial heterogeneity; models differentiation mechanisms	Elevated GI_50_ values indicate drug penetration barriers and lack vascular and immune components and are technically complex	[130]
Combination therapy models	Primary AML cells, AML PDX in NSG mice	BAY1436032 + AZA	In vitro: 80% colony inhibition with combination (CI <0.63–0.68 at ED_50_/_75_/_95_), indicating strong synergy. In vivo: Simultaneous combination reduced LSC frequency 33,150‐fold (vs. vehicle), 282‐fold greater than BAY1436032 alone; synergistic suppression of MAPK/ERK and Rb/E2F pathways	Reveals synergistic mechanisms for rational combination design; demonstrates profound LSC eradication; clinically relevant for AML combination therapy	LSC targeting complexity in clinical translation; lacks immune microenvironment modeling; requires optimization of dosing sequence	[129]
PK/PD modeling	Mice, rats, cynomolgus monkeys; dose‐ranging and exposure‐response studies	BAY1436032, vorasidenib	BAY1436032 150 mg/kg: linear exposure (45–150 mg/kg), unbound drug >IC_50_ for 24 h, near‐complete 2‐HG suppression. Vorasidenib 150 mg BID (human): *C*_max_ 1.2 µM, AUC 6.6 µM h, <1.5‐fold deviation from preclinical predictions	Enables dose and schedule optimization; validates translational fidelity for clinical dosing; correlates drug exposure with target engagement	Species differences require cross‐validation; human PK predictions need clinical confirmation; limited data on inter‐patient variability	[124, 127]
Resistance mechanism models	Relapsed AML PDX with single‐cell DNA sequencing; longitudinal clonal tracking	BAY1436032 + AZA combination	75% of relapsed cases (9/12) exhibited clonal evolution with RUNX1, ETV6, or NRAS mutations; clearance of IDH1^+^/NPM1^+^/NRAS^+^ clones and expansion of IDH1‐negative subclone; resistance not due to isoform switching or secondary IDH mutations	Reveals authentic resistance pathways and clonal dynamics; guides next‐generation therapeutic strategies; identifies non‐IDH escape mechanisms	High analytical and computational complexity; requires advanced single‐cell technologies; resource‐intensive with limited throughput	[29]

Abbreviations: 3D, three‐dimensional; AML, acute myeloid leukemia; AUC, area under the curve; AZA, azacitidine; BBB, blood–brain barrier; CI, combination index; CNS, central nervous system; D‐2HG, d‐2‐hydroxyglutarate; GI_50_, concentration causing 50% growth inhibition; IC_50_, half‐maximal inhibitory concentration; LSC, leukemic stem cell; NSG, NOD‐SCID‐gamma; PD, pharmacodynamic; PDX, patient‐derived xenograft; PK, pharmacokinetic; WT, wild‐type.

## 4 Clinical Advances: Development of Mutant IDH Inhibitors

Since the initial regulatory approval of enasidenib in 2017, the IDH inhibitor portfolio has expanded to include ivosidenib, olutasidenib, and vorasidenib, demonstrating therapeutic value across AML, CCA, and gliomas. Although monotherapy has achieved favorable response rates with manageable safety profiles, clinical experience has also revealed inherent limitations, including primary and acquired resistance, mutation‐specific efficacy patterns, and limited durability of responses.

### 4.1 Mutant IDH1 Inhibitors

#### 4.1.1 Ivosidenib (AG‐120)

Ivosidenib represents the inaugural success in targeted mIDH1 therapy. Developed through a pioneering drug discovery program by Agios Pharmaceuticals, it is a highly selective, orally bioavailable allosteric inhibitor. The molecule exerts its therapeutic effect by binding to the allosteric pocket of mutant IDH1 enzymes, stabilizing an inactive “open” conformation. This prevents the conformational shifts required for D‐2HG production and catalytic function [123]. This mechanism‐based approach directly targets the pathogenic driver in IDH1‐mutant cancers while sparing the WT IDH1 activity essential for normal cellular metabolism.

The FDA first granted approval for ivosidenib in July 2018 for adults with relapsed or refractory (R/R) AML harboring an IDH1 mutation [131]. This indication was subsequently expanded to include newly diagnosed AML in patients aged 75 or older or those with comorbidities that preclude intensive chemotherapy. Further approvals have extended their use to advanced or metastatic CCA with IDH1 mutations and, most recently, to R/R myelodysplastic syndromes (MDS) [132, 133]. The recommended therapeutic dose is 500 mg administered orally once daily until disease progression or intolerable toxicity [131].

Ivosidenib's clinical efficacy was established in a multicenter Phase I study (NCT02074839) of 258 patients with advanced hematologic malignancies. In 179 relapsed/refractory IDH1‐mutant AML patients treated with 500 mg daily, the composite complete remission (CR)/CRh rate was 30.4% (95% CI: 22.5–39.3), the CR rate was 21.6% (95% CI: 14.7–29.8), and the overall response rate (ORR) was 41.6% (95% CI: 32.9–50.8). Median response durations were 8.2 months (95% CI: 5.5–12.0), 9.3 months (95% CI: 5.6–18.3), and 6.5 months (95% CI: 4.6–9.3), respectively. Transfusion independence was achieved in 35% of evaluable patients (29/84). In 34 newly diagnosed patients ineligible for intensive chemotherapy, outcomes were notably improved: composite CR/CRh rate 42.4% (95% CI: 25.5–60.8), CR rate 30.3% (95% CI: 15.6–48.7), ORR 54.5% (95% CI: 36.4–71.9), with a median OS of 12.6 months (95% CI: 4.5–25.7) and a 1‐year OS rate of 51.1% [134, 135].

In the Phase I trial NCT02632708, evaluating ivosidenib in combination with intensive chemotherapy for newly diagnosed IDH1‐mutant AML, 60 patients were enrolled in the ivosidenib arm. The composite rate of CR plus CR with incomplete hematologic recovery (CRi) plus CR with incomplete platelet recovery (CRp) was 77%, with a CR rate of 68%. Response rates were higher in de novo AML compared to secondary AML. With a median follow‐up of 14.5 months, median OS was not reached (12‐month survival rate: 78%). These findings demonstrate robust efficacy of ivosidenib combined with intensive chemotherapy in newly diagnosed IDH1‐mutant AML, particularly in de novo cases, with 12‐month survival rates exceeding 70%, supporting this combination as a viable treatment option [136].

In the Phase III ClarIDHy trial (NCT02989857) evaluating ivosidenib in advanced IDH1‐mutant CCA, the agent demonstrated a statistically and clinically meaningful survival benefit after adjustment for crossover effects. In the intention‐to‐treat population, median OS was 10.8 months (95% CI: 7.7–17.6) in the ivosidenib arm versus 9.7 months (95% CI: 4.8–12.1) in the placebo arm, which did not reach statistical significance (HR 0.69, 95% CI: 0.44–1.10, one‐sided *p* = 0.060). However, when accounting for the confounding effect of crossover, where 57% of placebo patients received ivosidenib following disease progression, rank‐preserving structural failure time analysis estimated the true placebo median OS at only 6.0 months (95% CI: 3.6–6.3). This revealed a 54% reduction in risk of death for ivosidenib versus placebo (HR 0.46, 95% CI: 0.28–0.75, one‐sided *p* < 0.001), thereby confirming the OS benefit of ivosidenib. Concurrently, median progression‐free survival (PFS) was significantly longer in the ivosidenib arm compared to placebo (2.7 vs. 1.4 months, HR 0.37, 95% CI: 0.25–0.54, one‐sided *p* < 0.001). Additionally, ivosidenib delayed deterioration of physical function with a favorable safety profile and no treatment‐related deaths [137].

Final OS analysis revealed a median OS of 10.3 months (95% CI: 7.8–12.4) in the ivosidenib arm versus 7.5 months (95% CI: 4.8–11.1) in the placebo arm, which did not reach statistical significance (HR 0.79, one‐sided *p* = 0.09). However, after adjusting for crossover effects using the rank‐preserving structural failure time model, the true placebo median OS was reduced to 5.1 months (95% CI: 3.8–7.6), demonstrating a 51% reduction in risk of death with ivosidenib (HR 0.49, 95% CI: 0.34–0.70, one‐sided *p* < 0.001). This represents breakthrough efficacy in this historically poor‐prognosis, aggressive malignancy [138].

In the glioma cohort (NCT02073994), ivosidenib demonstrated disease‐stabilizing activity with acceptable tolerability. Specifically, 85.7% (30/35) of patients with non‐enhancing IDH1‐mutant gliomas (including astrocytoma and oligodendroglioma subtypes) achieved stable disease (SD), with tumor volume reduction from baseline observed in 66.7% (22/33) of patients with measurable tumors. In contrast, 45.2% (14/31) of patients with enhancing IDH1‐mutant gliomas achieved SD. mPFS was significantly longer in the non‐enhancing cohort at 13.6 months (95% CI: 9.2–33.2) compared to 1.4 months (95% CI: 1.0–1.9) in the enhancing cohort, reflecting extended progression‐free intervals in a subset of patients. However, only one patient with non‐enhancing glioma achieved partial response (PR) across the entire cohort, with no CR observed. The rarity of complete responses in the solid tumor setting may reflect differences in TME accessibility and mutational complexity compared to hematologic malignancies [139].

The safety profile of ivosidenib has proven generally manageable across diverse patient populations. In the pivotal AML study, treatment‐emergent adverse events (TEAEs) occurred universally, with 79% of patients experiencing at least one Grade 3 or higher event [134]. The most clinically significant drug‐related toxicities included QT interval prolongation on electrocardiography (7.8%), IDH differentiation syndrome (3.9%), and anemia (2.2%). Differentiation syndrome, a potentially life‐threatening complication initially characterized in acute promyelocytic leukemia treated with all‐trans retinoic acid (ATRA) [140], manifests through capillary leak‐mediated edema, hypotension, acute respiratory distress, and febrile leukocytosis. Prompt recognition and initiation of corticosteroid therapy effectively manage this syndrome in the majority of cases, with cytoreduction reserved for concomitant hyperleukocytosis [141]. In the CCA trial, the safety profile proved even more favorable, with only three patients (1.6%) discontinuing ivosidenib owing to treatment‐related adverse events—specifically Grade 3–4 hyperbilirubinemia, Grade 2 QT prolongation, and Grade 3 pleural effusion—and without treatment‐related deaths [137, 138].

Pharmacokinetic studies have characterized ivosidenib as exhibiting rapid oral absorption with low systemic clearance, resulting in accumulation to steady‐state concentrations capable of sustaining >95% suppression of plasma D‐2HG levels throughout the dosing interval [142]. This pharmacodynamic effect directly correlates with target engagement and serves as a validated biomarker for on‐target activity. The favorable PK/PD profile supports once‐daily oral dosing and contributes to the drug's clinical utility in outpatient management of chronic conditions.

#### 4.1.2 Olutasidenib (FT‐2102): Second‐Generation IDH1 Inhibitor

Olutasidenib emerged from Forma Therapeutics’ structure‐guided drug design efforts as a potent and selective oral IDH1 inhibitor with differentiated molecular properties. The FDA granted accelerated approval in December 2022 for adult patients with R/R AML harboring susceptible IDH1 mutations, positioning olutasidenib as the second approved agent in this therapeutic class [18].

Structural studies reveal that olutasidenib occupies a hydrophobic allosteric pocket proximal to the IDH1 homodimer interface. It engages the enzyme via an additional hydrogen bond with Ile128, distinguishing its binding mode from that of ivosidenib [143]. Notably, olutasidenib exhibits a 2:1 stoichiometry (two inhibitor molecules per IDH1 dimer), contrasting with the 1:1 ratio of ivosidenib. This structural distinction may contribute to its broad‐spectrum activity against multiple IDH1 R132 variants, including R132H, R132C, R132G, and R132L [144].

Regulatory approval was based on a pivotal Phase I/2 trial (NCT02719574) enrolling 153 patients with R/R IDH1‐mutant AML. Following Phase I dose optimization (150 mg twice daily), the Phase II expansion cohort enrolled 147 evaluable patients treated until disease progression, prohibitive toxicity, or hematopoietic stem cell transplantation [145].

Treatment with olutasidenib induced a median 82% reduction in plasma D‐2HG concentrations after two cycles of treatment (with an 83% reduction in patients who achieved CR/CRh and an 80% reduction in other responders), and this reduction was sustained throughout subsequent treatment cycles, validating robust target engagement. The CR/CRh rate was 35% (95% CI: 27.0–43.0), with a CR rate of 32% (95% CI: 24.5–40.2) and an ORR of 48% (95% CI: 40.0–56.7). These rates compare favorably to those of ivosidenib and other IDH1 inhibitors, supporting olutasidenib's positioning as an effective therapeutic option.

Outcomes in the venetoclax‐pretreated subgroup were notably robust, despite the challenge of prior BCL‐2 inhibitor exposure. The CR/CRh rate was 33% (95% CI: 9.9–65.1), and the CR rate was 25% (95% CI: 5.5–57.2). Two patients achieved CRi and two achieved SD, resulting in an ORR of 50% (*n* = 6; 95% CI: 21.1–78.9). Clinically, two of the three patients achieving CR and one achieving CRh remained in remission and continued treatment at the data cutoff. These results demonstrate that olutasidenib retains activity in R/R IDH1‐mutant AML after prior venetoclax exposure [145].

The safety profile was consistent with class‐effect toxicities. Grade 3 or higher TEAEs included febrile neutropenia (20%), anemia (20%), thrombocytopenia (16%), and neutropenia (13%). Differentiation syndrome occurred in 14% of patients, hepatic adverse events of special interest in 25%, and QT interval prolongation in 8%. The adverse event spectrum of olutasidenib monotherapy was manageable, with most events addressed through dose modifications or standard supportive care [145].

The approval of olutasidenib as monotherapy for relapsed/refractory AML expands the therapeutic armamentarium for IDH1‐mutant disease and provides an alternative option for patients who may develop resistance to or experience intolerance of ivosidenib. Ongoing studies are exploring olutasidenib's activity in additional IDH1‐mutant malignancies and evaluating optimal integration into treatment algorithms.

#### 4.1.3 BAY1436032: Investigational IDH1 Inhibitor With Stem Cell Activity

BAY1436032 represents a collaborative development between Bayer and the German Cancer Research Center, engineered as an orally bioavailable small molecule inhibitor targeting diverse IDH1 mutation variants. The compound demonstrated several distinctive biological properties that differentiated it from contemporary IDH1 inhibitors, including particularly potent suppression of leukemic stem cell self‐renewal capacity. In secondary transplantation experiments comparing BAY1436032 to ivosidenib, mice treated with BAY1436032 exhibited markedly reduced LSC frequency, suggesting superior eradication of the treatment‐resistant stem cell compartment that drives disease relapse [129]. This finding suggested potential for enhanced durability of clinical responses through more complete elimination of the leukemic clone hierarchy.

Despite these encouraging biological features, clinical translation proved disappointing. A Phase I dose‐escalation trial in patients with IDH1‐mutant AML (NCT03127735) revealed suboptimal efficacy, with an ORR of only 15% and median OS of 6.6 months (95% CI: 4.6–9.4), outcomes substantially inferior to ivosidenib and olutasidenib [146]. Pharmacodynamic assessments demonstrated reduction in baseline D‐2HG levels across all treated patients; however, normalization to physiologic ranges occurred in only 5 of 26 evaluable patients (19%), indicating incomplete target suppression even at maximal administered doses. The safety profile proved acceptable, with differentiation syndrome affecting 19% of participants and no Grade 3 or higher QT interval prolongation events. However, the fundamental limitation of insufficient target engagement and correspondingly modest clinical activity led to discontinuation of further AML development.

Parallel evaluation in solid tumors yielded mixed results. A Phase I trial (NCT02746081) enrolling 71 patients with diverse IDH1‐mutant solid malignancies reported an ORR of 6% and SD rate of 41%, with 3‐month PFS probability of 25% (95% CI: 15–35) [147]. LGG patients demonstrated the most favorable outcomes, representing the only tumor type achieving objective remissions (ORR 11% in LGG cohort). In CCA patients, BAY1436032 induced rapid and sustained reductions in plasma D‐2HG, with median maximal decline of 76%, achieving concentrations approximating those in healthy individuals. The solid tumor safety profile proved highly tolerable, with predominantly Grade 1–2 adverse events in the dose‐escalation cohort and only one Grade 4 drug‐related event (lipase elevation) in the expansion cohort, which resolved following drug discontinuation [147].

The clinical development trajectory of BAY1436032 underscores the challenges inherent in translating preclinical promises to clinical efficacy, particularly when insufficient pharmacokinetic properties or tissue distribution limit achievement of therapeutic drug concentrations at the target site. Although the compound contributed valuable biological insights regarding leukemic stem cell targeting, its limited clinical activity relative to approved IDH1 inhibitors precluded further advancement in competitive therapeutic landscapes.

#### 4.1.4 DS‐1001b: Brain‐Penetrant IDH1 Inhibitor for CNS Malignancies

DS‐1001b emerged from the medicinal chemistry program of Daiichi Sankyo with a specific objective: achieving sufficient BBB penetration to enable effective targeting of IDH1‐mutant gliomas, a population inadequately served by first‐generation IDH inhibitors with limited CNS exposure. The molecule exhibits selectivity for IDH1 R132H, the predominant mutation variant in gliomas, and was optimized for physicochemical properties favoring CNS penetration [126].

A Phase I trial (NCT04458272) evaluating DS‐1001b in patients with IDH1‐mutant glioma revealed differential activity based on tumor enhancement characteristics on magnetic resonance imaging, reflecting underlying biological heterogeneity [148]. Among 47 patients with contrast‐enhancing tumors, the ORR reached 17.1%, with two patients achieving CR, a rare outcome in recurrent glioblastoma. The mPFS in this cohort was 10.4 weeks (95% CI: 6.1–17.7 weeks). In contrast, patients with non‐enhancing tumors demonstrated substantially more favorable responses, with an ORR of 33.3% and median PFS not yet reached (95% CI: 24.1 weeks–not reached) at data cutoff. Reductions in tumor measurements from baseline occurred in 15 of 35 patients (43%) with enhancing disease and 11 of 12 patients (92%) with non‐enhancing disease. Pharmacodynamic validation was provided by significant D‐2HG reductions in brain tumor tissue samples from seven patients with paired pre‐ and on‐treatment biopsies.

The contrasting efficacy profiles between enhancing and non‐enhancing gliomas likely reflect fundamental differences in tumor biology rather than drug‐specific limitations. Enhancing gliomas typically contain additional genetic alterations beyond IDH1 mutation, including CDKN2A/B deletions and TP53 mutations, which confer aggressive growth characteristics that may circumvent dependence on mutant IDH1 as an oncogenic driver. Non‐enhancing lower grade gliomas more closely resemble IDH‐mutant tumors at earlier evolutionary stages, where mutant IDH1 remains the dominant oncogenic driver and cells retain greater differentiation capacity.

The safety profile of DS‐1001b was highly manageable, with predominant adverse events consisting of Grade 1–2 dermatologic manifestations, including skin pigmentation changes, pruritus, and alopecia, along with gastrointestinal symptoms (diarrhea, nausea), arthralgia, headache, and rash [148]. Favorable tolerability supports chronic administration, which is essential in glioma populations where disease control over extended periods represents the primary therapeutic objective.

Two Phase II trials are currently evaluating the single‐agent activity of DS‐1001b: one (NCT04458272) in chemotherapy‐ and radiotherapy‐naïve patients with IDH1‐mutant WHO Grade 2 gliomas and another (NCT05303519) in recurrent or progressive IDH1‐mutant WHO Grade 2/3 gliomas. These studies will provide definitive efficacy data in homogeneous patient populations and clarify the optimal positioning of DS‐1001b within glioma treatment algorithms.

#### 4.1.5 IDH305: Early‐Generation IDH1 Inhibitor With Limited Development

IDH305, developed by Novartis, entered clinical evaluation as an orally bioavailable selective IDH1 inhibitor with rapid absorption kinetics. A Phase I trial (NCT02381886) in patients with IDH1‐mutant AML and MDS demonstrated target engagement, with D‐2HG concentration reductions observed in 85.4% of patients across all dose levels [149]. Clinical activity was evidenced by CRs in 10 of 37 patients with AML (27%) and one of four patients with MDS (25%).

However, the development program encountered significant hepatotoxicity concerns that ultimately led to early termination. All patients experienced at least one adverse event during the study period, with events suspected to be drug‐related reported by 53.7%. The most concerning toxicities included elevated blood bilirubin (14.6%), nausea (14.6%), elevated alanine aminotransferase (12.2%), and elevated aspartate aminotransferase (12.2%) [149]. Although the hepatotoxicity proved reversible upon drug discontinuation, investigators concluded that the therapeutic window of IDH305, the margin between efficacious and toxic doses, was insufficiently wide to support further development in competitive landscapes where alternative IDH1 inhibitors (ivosidenib and olutasidenib) demonstrated superior safety profiles. This outcome underscores the critical importance of comprehensive toxicology assessment during early‐phase drug development and the decisions regarding clinical advancement.

#### 4.1.6 LY3410738: Covalent Second‐Generation IDH1 Inhibitor

LY3410738 represents the entry of Eli Lilly into IDH1 inhibitor development, distinguished as a first‐in‐class covalent inhibitor classified as a second‐generation agent [38]. Unlike reversible allosteric inhibitors such as ivosidenib and olutasidenib, which bind noncovalently within the dimerization interface, LY3410738 employs a covalent binding mechanism targeting a distinct site outside the classical allosteric pocket. This alternative binding mode confers several theoretical advantages, including enhanced binding affinity for IDH1 R132H mutants, prolonged target engagement through irreversible covalent modification, and most significantly, retained activity against secondary resistance mutations that emerge at the canonical allosteric site during treatment with first‐generation inhibitors.

A Phase I dose‐escalation trial (NCT04603001) in patients with R/R IDH1/2‐mutant hematologic malignancies provided preliminary safety and pharmacodynamic data. The median treatment duration was 2.3 months, with common TEAEs including diarrhea (22%), fatigue (21%), and anemia (20%). Differentiation syndrome developed in 10% of patients, with 6% experiencing Grade 3 severity [38]. Pharmacokinetic analyses demonstrated dose‐dependent drug exposure, and pharmacodynamic assessments confirmed sustained D‐2HG suppression across all dose levels, validating target engagement. However, efficacy appeared diminished in patients who had previously received IDH inhibitor therapy, suggesting potential for cross‐resistance despite the distinct binding mechanism, a finding warranting further mechanistic investigation.

An additional Phase I trial (NCT04521686) evaluating LY3410738 in 119 patients with IDH‐mutant solid tumors demonstrated rapid and durable D‐2HG suppression but largely cytostatic single‐agent activity, with ORR of only 5.2% and 11.1% in relapsed/refractory CCA and glioma, respectively. In contrast, combination with cisplatin‐gemcitabine in treatment‐naive IDH‐mutant CCA achieved a 42.1% response rate with median PFS of 10.2 months, suggesting that chemotherapy combinations may overcome the limited efficacy of IDH inhibitor monotherapy in solid tumors.

### 4.2 Mutant IDH2 Inhibitors

#### 4.2.1 Enasidenib (AG‐221, IDHIFA): Pioneering IDH2‐Selective Therapy

Enasidenib was the first FDA‐approved selective inhibitor of mutant IDH2, receiving regulatory authorization in August 2017 for the treatment of R/R AML harboring IDH2 mutations [16]. Developed by Celgene Corporation, enasidenib functions as an orally bioavailable allosteric inhibitor that binds to the dimer interface of mutant IDH2 enzymes, inducing conformational changes that block D‐2HG production while restoring normal cellular differentiation programs. The approved dosing regimen consists of 100 mg administered orally once daily on a continuous schedule until disease progression or unacceptable toxicity.

The pivotal registration trial (AG221‐C‐001, NCT01915498) enrolled 239 patients with IDH2‐mutant AML or MDS across dose‐escalation (50–650 mg) and expansion phases, with primary efficacy analysis focused on 176 patients with R/R AML receiving the 100 mg daily dose [16]. The CR rate reached 19.3% (95% CI: 13.8–25.9), with an ORR of 40.3% (95% CI: 33.0–48.0) and median OS of 9.3 months (95% CI: 8.2–10.9 months). Among patients treated at the recommended 100 mg dose, the CR rate was 20.2% (95% CI: 13.1–28.9) and the ORR was 38.5% (95% CI: 29.4–48.3). Notably, the magnitude of D‐2HG suppression demonstrated mutation‐specific patterns, with IDH2 R140 mutations showing more profound reductions compared to R172 variants. The degree of D‐2HG suppression strongly correlated with the probability of achieving CR, providing validation of the mechanistic hypothesis linking D‐2HG reduction to therapeutic efficacy. The cohort of 17 patients with MDS demonstrated an ORR of 53% (95% CI: 28–77), suggesting activity across the spectrum of IDH2‐mutant myeloid neoplasms [150].

Safety analyses revealed a manageable toxicity profile characterized predominantly by indirect hyperbilirubinemia (38% of patients), nausea (23%), and differentiation syndrome (6%) [16, 151]. The hyperbilirubinemia was generally mild and asymptomatic, attributed to enasidenib's inhibition of UDP‐glucuronosyltransferase 1A1 (UGT1A1)‐mediated bilirubin glucuronidation, and rarely necessitated dose modifications. Differentiation syndrome, while potentially serious, responded consistently to corticosteroid therapy when recognized promptly. Grade 3 or higher adverse events in the MDS cohort included indirect hyperbilirubinemia (35%), pneumonia (29%), thrombocytopenia (24%), and differentiation syndrome (18%) [150].

A subsequent Phase III randomized trial (NCT02577406) compared enasidenib to conventional care regimens (AZA, intermediate‐dose cytarabine, low‐dose cytarabine, or best supportive care) in 319 patients with R/R IDH2‐mutant AML who were preselected for their intended conventional therapy prior to randomization [152]. Although median OS did not differ significantly between enasidenib (6.5 months) and conventional care (6.2 months), enasidenib demonstrated substantially superior response rates: ORR of 40.5% versus 9.9%, composite CR rate (CR + CRi + CRp) of 29.7% versus 6.2%, and CR/CRh rate of 25.3% versus 5.0%. Additionally, enasidenib showed significant advantages across multiple secondary endpoints: event‐free survival was significantly prolonged (4.9 vs. 2.6 months), time to treatment failure was notably improved (4.9 vs. 1.9 months), and enasidenib demonstrated superior hematologic improvement and achievement of red blood cell transfusion independence compared to conventional care.

The disconnect between improved response rates and lack of OS benefit likely reflects the innovative preselection design of the trial, which allowed crossover to enasidenib following progression on conventional therapy, and the aggressive nature of multiply relapsed AML in older populations with limited tolerance for subsequent salvage therapies. Treatment‐related adverse events included nausea, hyperbilirubinemia, and thrombocytopenia, with hyperbilirubinemia representing the only common Grade 3 or higher toxicity, and differentiation syndrome occurring in 5.1% of enasidenib‐treated patients [152].

#### 4.2.2 Vorasidenib (AG‐881, Voranigo): Dual IDH1/2 Inhibitor With CNS Penetration

Vorasidenib represents a pharmacological innovation as the first brain‐penetrant dual inhibitor capable of potently targeting both mutant IDH1 and IDH2 isoforms through a single molecular entity. Developed by Agios Pharmaceuticals through systematic medicinal chemistry optimization, vorasidenib was intentionally designed to fulfill stringent physicochemical criteria enabling BBB penetration while maintaining high‐affinity binding to both mIDH variants [82, 127]. The molecule exhibits symmetric binding within the allosteric pockets of both mutant IDH1‐R132H and IDH2‐R140Q enzymes, engaging conserved residues through hydrogen bonding and halogen interactions while the trifluoromethyl moiety establishes van der Waals contacts with hydrophobic residues to stabilize the catalytically inactive open conformation.

The FDA granted approval for vorasidenib on August 6, 2024, for the treatment of adults and pediatric patients aged 12 years and older with Grade 2 astrocytoma or oligodendroglioma harboring susceptible IDH1 or IDH2 mutations following surgical resection. The approved dosing regimen consists of 40 mg orally once daily for adults and patients weighing ≥40 kg, with dose reduction to 20 mg daily for pediatric patients weighing <40 kg. This approval marked the first IDH‐targeted therapy specifically indicated for lower grade gliomas, representing a paradigm shift in the management of these indolent yet ultimately progressive malignancies.

The regulatory decision was based on the landmark INDIGO Phase III trial (NCT04164901), a multicenter, randomized, double‐blind, placebo‐controlled study enrolling 331 patients with residual or recurrent IDH1/2‐mutant Grade 2 gliomas who had undergone surgical resection without prior chemotherapy or radiotherapy [153]. Patients were randomized 1:1 to vorasidenib (*n* = 168) or placebo (*n* = 163), stratifying by histology (astrocytoma vs. oligodendroglioma) and enhancing disease status.

Vorasidenib treatment resulted in profoundly prolonged PFS compared to placebo, with median PFS of 27.7 months (95% CI: 17.0–not estimable) versus 11.1 months (95% CI: 11.0–13.7), corresponding to a 61% reduction in progression or death risk (HR 0.39, 95% CI: 0.27–0.56, *p* < 0.001). The benefit was consistent across prespecified subgroups defined by histology, mutation type, and enhancement status. Additionally, vorasidenib significantly prolonged time to next intervention, a clinically meaningful endpoint capturing when patients require subsequent therapy such as radiotherapy or chemotherapy, with median time to next intervention not yet reached in the vorasidenib arm versus 17.8 months in the placebo arm, representing a 74% reduction in risk of requiring subsequent treatment.

The safety profile was acceptable for chronic administration in a patient population with relatively preserved neurological function and quality of life at trial entry. Grade 3 or higher adverse events occurred in 22.8% of vorasidenib‐treated patients, with alanine aminotransferase elevation representing the most common serious toxicity. Grade 3–4 laboratory abnormalities included elevated ALT, AST, gamma‐glutamyl transferase, and neutropenia [153]. Importantly, the transaminase elevations were reversible with dose interruption or reduction and did not result in cases of hepatic failure or irreversible injury. No patients developed clinically significant QT interval prolongation, and differentiation syndrome, a concern in myeloid malignancies, did not occur in the solid tumor setting.

Vorasidenib has also undergone evaluation in hematologic malignancies, though with less favorable outcomes. A Phase I trial (NCT02492737) enrolled 46 patients with IDH1/2‐mutant myeloid neoplasms (34 AML, 11 MDS, and 1 angioimmunoblastic T‐cell lymphoma) [154]. A total of 35 of 46 (76.1%) patients with AML had been treated with prior enasidenib or ivosidenib, creating a heavily pretreated, potentially cross‐resistant population. ORRs were modest: 5.9% (95% CI: 0.7–19.7) in AML and 36.4% (95% CI: 10.9–69.2) in MDS. The superior activity in patients with MDS, most of whom were naive to IDH inhibitors, contrasted with the poor outcomes in patients with AML who were previously treated, suggesting either disease biology differences or acquired resistance mechanisms. Plasma 2‐HG reductions were more pronounced in IDH inhibitor‐naive patients with MDS compared to patients with AML with prior IDH inhibitor exposure, supporting a role for treatment history in pharmacodynamic response. These findings, combined with a suboptimal dose‐efficacy relationship in the pretreated AML population and the limited efficacy observed in patients previously treated with mIDH inhibitors, have led to the discontinuation of vorasidenib development in hematologic malignancies. The program is now focused exclusively on gliomas, where its brain penetration properties confer a unique therapeutic advantage [154, 155].

#### 4.2.3 Ranosidenib (HMPL‐306): A Novel Dual IDH1/2 Inhibitor

HMPL‐306 is a new potent and selective dual inhibitor of mutant IDH1 and 2. This compound exhibits favorable PK properties with a low risk of hERG channel inhibition and off‐target effects. Preclinical studies demonstrate that HMPL‐306 significantly and durably suppresses 2‐HG levels in mIDH1‐ and mIDH2‐driven tumor xenograft models and displays high brain penetrance in mice. Notably, a head‐to‐head in vivo comparison conducted by Xiao et al. revealed that HMPL‐306 achieves superior brain exposure relative to vorasidenib (AG‐881) [156]. HMPL‐306 is currently under clinical evaluation and has demonstrated a favorable safety profile and encouraging preliminary efficacy in patients with IDH‐mutant AML [157]. Through iterative structure–activity relationship studies and drug metabolism and pharmacokinetics optimization, HMPL‐306 was developed as a novel orally bioavailable small‐molecule dual inhibitor of mIDH1 and mIDH2. Preclinical characterization demonstrated nanomolar cellular inhibitory IC_50_ values against both mIDH1 and mIDH2. In IDH‐mutant tumor xenograft models, a single oral dose of 25 mg/kg achieved ≥90% suppression of intratumoral 2‐HG, with the effect sustained for more than 40 h post‐administration. Furthermore, HMPL‐306 exhibited low systemic clearance, a prolonged half‐life, and high oral bioavailability. Compared with approved mIDH inhibitors, its off‐target inhibition of hERG channels and UGT1A1 was substantially attenuated, conferring a more favorable therapeutic window [156].

A first‐in‐human Phase I trial enrolled 76 Chinese patients with R/R mIDH1/2‐mutant AML across multiple centers. No dose‐limiting toxicities were observed, and the recommended Phase II (RP2D) dose was established as 250 mg once daily during Cycle 1 (loading dose), followed by 150 mg once daily from Cycle 2 onwards (maintenance dose). Safety analyses revealed that the incidences of differentiation syndrome and QTc prolongation were markedly lower than those reported with approved single‐target IDH inhibitors, and only 3.9% of patients required permanent treatment discontinuation due to adverse events. In terms of efficacy, among patients treated at the RP2D (*n* = 59), the composite CR rate was 34.6% and 36.4% in the mIDH1 and mIDH2 subgroups, respectively, with a substantial proportion of patients achieving measurable residual disease (MRD)‐negative deep remission. The median OS exceeded 13 months in both subgroups. Collectively, these findings support HMPL‐306 as a promising novel therapeutic strategy targeting mIDH1/2, with the potential to overcome acquired resistance observed following treatment with single‐isoform IDH inhibitors, thereby offering improved outcomes for patients with R/R AML. A Phase III randomized controlled registration trial, RAPHAEL (NCT06387069), has been formally initiated and is anticipated to further establish HMPL‐306 as a new standard‐of‐care option for this patient population.

### 4.3 Summary and Clinical Implications

The clinical development of mutant IDH inhibitors represents a landmark achievement in precision oncology, successfully translating mechanistic insights into FDA‐approved targeted therapies (Table 3). Ivosidenib, olutasidenib, enasidenib, and vorasidenib have demonstrated consistent efficacy in suppressing 2‐HG production and restoring cellular differentiation across AML, MDS, CCA, and gliomas, achieving response rates of 20%–50% with manageable safety profiles. However, clinical experience reveals important limitations: modest absolute response rates, disease‐context dependencies (mutation variants, tumor types, prior therapies), and disease progression in most patients. The emergence of diverse resistance mechanisms, including second‐site mutations, clonal evolution, pathway activation, and metabolic reprogramming, represents the most formidable challenge limiting therapeutic durability, necessitating rational combination strategies to achieve sustained disease control.

**TABLE 3:** Clinical development of mutant isocitrate dehydrogenase (mIDH) inhibitors.

Drug	Trial ID	Cancer type/subtype	Study design	Study period/sample size	Key clinical outcomes	Reference
Ivosidenib	NCT02074839	Relapsed/Refractory (R/R) IDH1‐mutant AML	Phase I, open‐label, non‐randomized, Single‐arm	2014–2017/*n* = 258	ORR: 41.6%; CR: 21.6%; mOS: 8.8 months; mDoR: 9.3 months	[135]
Ivosidenib + azacitidine (AZA)	NCT03173248 (AGILE trial)	Newly diagnosed IDH1‐mutant AML (≥75 years or ineligible for intensive chemotherapy)	Phase III, randomized, double‐blind, placebo‐controlled	2017–2021/*n* = 146	(ivosidenib + AZA) vs. (placebo + AZA): month EFS rate: 37% vs. 12%; OS: 24 vs. 7.9 months; CR: 47% vs. 15%; ORR: 63% vs. 19%	[24]
Ivosidenib	NCT02989857 (ClarIDHy trial)	Previously treated IDH1‐mutant CCA	Phase III, randomized, double‐blind, placebo‐controlled	2017–2019/*n* = 185	Ivosidenib vs. placebo: mPFS: 2.7 vs. 1.4 months; mOS: 10.8 vs. 9.7 months; ORR: 2% vs. 0%; mPFS: 2.7 vs. 1.4 months	[137]
Ivosidenib	NCT02073994	IDH1‐mutated advanced glioma	Phase III, open‐label, non‐randomized, single‐arm	2014–2019/*n* = 168	Non‐enhancing glioma vs. enhancing glioma: ORR: 2.9% vs. 0%; mPFS: 13.6 vs. 1.4 months; median duration of treatment: 18.4 vs. 1.9	[139]
Ivosidenib	NCT02632708	Newly diagnosed IDH1‐mutant AML	Phase I, open‐label, non‐randomized, single‐arm	2016–2018/*n* = 60	CR: 68%; CR + CRi/CRp rate: 77%; the 12‐month OS rate: 78%; rate of IDH mutation clearance: 39%	[136]
Enasidenib	NCT02632708	Newly diagnosed IDH2‐mutant AML	Phase I, open‐label, non‐randomized, single‐arm	2016–2018/*n* = 91	CR: 55%; CR + CRi/CRp rate: 74%; mOS: 25.6 months; the 12‐month OS rate: 76%; rate of IDH mutation clearance: 23%	[136]
Olutasidenib	NCT02719574	Newly diagnosed IDH2‐mutant AML	Phase I, open‐label, non‐randomized, single‐arm	2016–2018/*n* = 153	CR: 32%; ORR: 48%; mOS: 11.6 months	[158]
Enasidenib	NCT01915498	Relapsed/Refractory IDH2‐mutant AML	Phase I/II, open‐label, non‐randomized, single‐arm	2013–2016/*n* = 239	ORR: 43%; CR: 19.3%; DCR: 88.6%; mDoR: 5.8 months	[16]
Enasidenib	NCT02577406	Relapsed/Refractory IDH2‐mutant AML	Phase III, open‐label, randomized	2016–2020/*n* = 319	Enasidenib vs. control: mOS: 6.5 vs. 6.2 months, 1‐year OS rate: 37.5% vs. 26.1%; mEFS: 4.9 vs. 2.6 months; ORR: 40.5% vs. 9.9%; composite CR rate (CR + CRi + CRp): 29.7% vs. 6.2%	[152]
Vorasidenib	NCT04164901 (INDIGO trial)	Low‐grade IDH2‐mutant glioma	Phase III, randomized, double‐blind, placebo‐controlled	2020–2022/*n* = 331	Vorasidenib vs. control: mPFS: 27.7 vs. 11.1 months; 24 months PFS rate: 62% vs. 21%	[153]

Abbreviations: AML, acute myeloid leukemia; AZA, azacitidine; CR, complete remission; CRh, complete remission with partial hematologic recovery; CRi, complete remission with incomplete count recovery; CRp, complete remission with incomplete platelet recovery; DCR, disease control rate; DoR, duration of response; EFS, event‐free survival; IDH, isocitrate dehydrogenase; ORR, overall response rate; OS, overall survival; PFS, progression‐free survival; R/R, relapsed/refractory.

## 5 Mechanistic Basis of Resistance to Mutant IDH Inhibitors

Resistance to mIDH inhibitors can be broadly categorized as primary resistance, which is present prior to treatment initiation, and acquired resistance, which develops during therapy. Both genetic and nongenetic mechanisms contribute to treatment failure, including disruption of drug‐target interactions, clonal evolution that selectively promotes the proliferation of resistant subpopulations, and adaptive metabolic reprogramming that sustains oncogenic signaling. Understanding the diverse resistance mechanisms is crucial for developing rational combination therapy strategies and next‐generation mIDH‐targeted therapeutics.

### 5.1 Molecular and Cellular Mechanisms of Resistance

**FIGURE 4:**
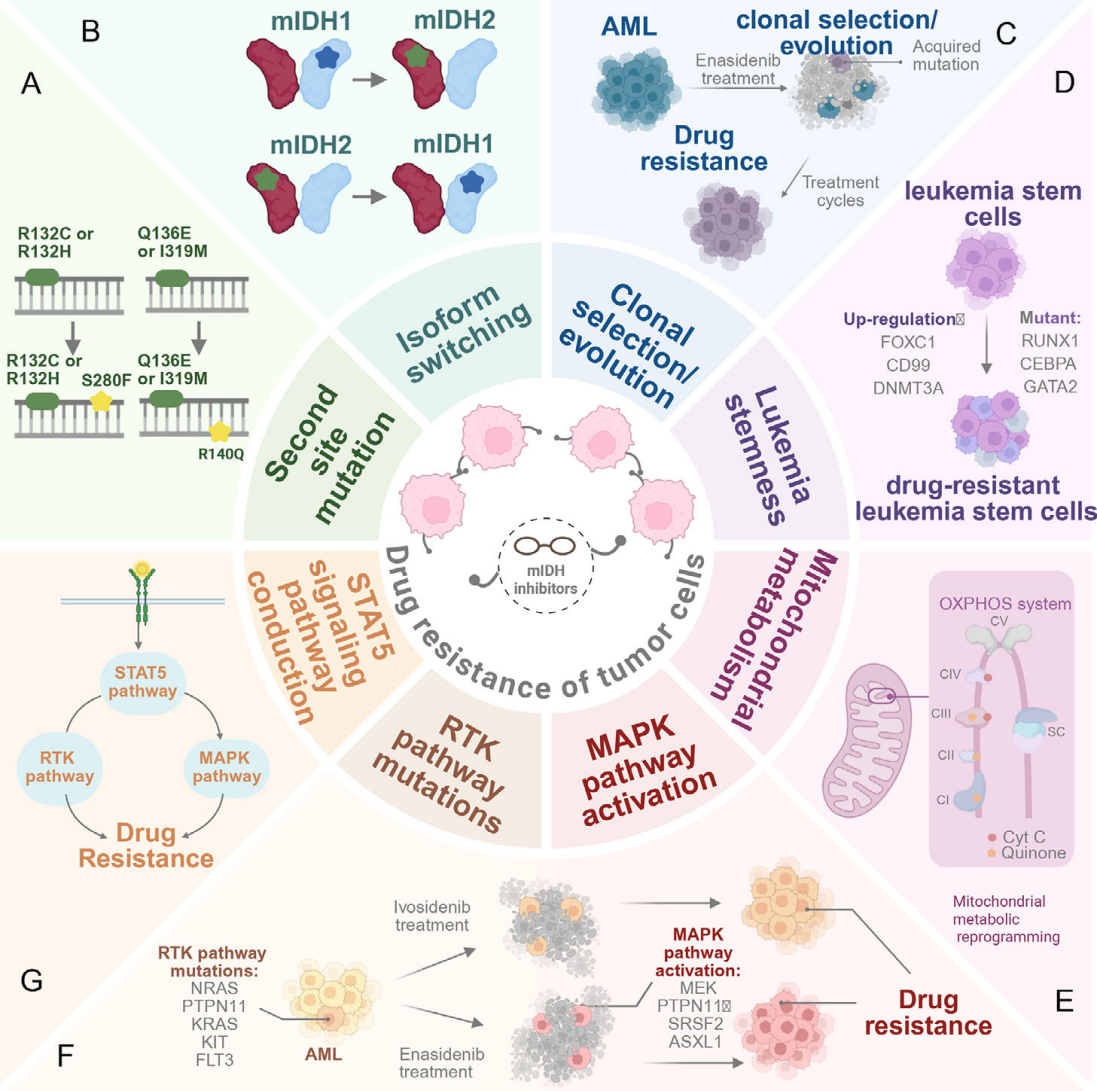
Mechanisms of resistance to mIDH inhibitors in cancer. Multiple mechanisms and physiological processes have a role in cancer resistance to mIDH inhibitors: (A) second site mutations, (B) isoform switching, (C) clonal selection/evolution, (D) leukemia stemness, (E) mitochondrial metabolism, (F) mitogen‐activated protein kinase (MAPK) pathway activation, receptor tyrosine kinase (RTK) pathway activation, (G) signal transducer and activator of transcription 5 (STAT5) signaling pathway conduction. AML, acute myeloid leukemia; ASXL1, additional sex comb‐like 1; CEBPA, CCAAT enhancer binding protein alpha; DNMT1, DNA methyltransferase 1; FLT3, fms‐like tyrosine kinase 3; FOXC1, forkhead box C1; MAPK, mitogen‐activated protein kinase; MEK, mitogen‐activated extracellular signal‐regulated kinase; mIDH, mutant isocitrate dehydrogenase; NRAS, neuroblastoma RAS viral oncogene homolog; OXPHOS, oxidative phosphorylation; PTPN11, protein tyrosine phosphatase non‐receptor type 11; RTK, receptor tyrosine kinase; RUNX1, runt‐related transcription factor 1; SRSF2, serine/arginine‐rich splicing factor 2.

Clinical studies have demonstrated the efficacy of mIDH inhibitors. However, primary and secondary resistances limit their durable clinical benefit. Cancer therapeutic resistance is a multifactorial process that is influenced not only by tumor‐intrinsic characteristics but also by the complex interplay between malignant cells and TME. It typically involves metabolic activity and extracellular metabolites, leading to metabolic crosstalk that serves not only as a source of bioenergetics support but also as a signaling modality facilitating intercompartmental communication. Multiple mechanisms underlying resistance to mIDH inhibitors have been identified to date (Figure 4) [159]. A comprehensive elucidation of the molecular underpinnings of resistance—including both tumor cell–intrinsic mechanisms and TME‐mediated extrinsic determinants—is critical for the development of immunotherapy‐associated targets and for the design of therapeutic strategies aimed at overcoming treatment resistance.

#### 5.1.1 Second Site Mutations and Isoform Switching

The first category of resistance to mIDH inhibitors arises from second‐site mutations in IDH1 or IDH2, which increase D‐2HG production and disrupt inhibitor binding, thereby leading to acquired resistance. In a study of two patients with mIDH2 AML treated with enasidenib, Intlekofer et al. identified resistance‐associated second‐site IDH2 mutations occurring on the trans allele [37]. Specifically, glutamine 316 to glutamate (Q316E) and isoleucine 319 to methionine (I319M), when combined with the original R140Q mutation, increase D‐2HG production and alter the enasidenib‐binding site at the dimerization interface, resulting in a loss of efficacy. Notably, the Q316E and I319M mutations alone do not affect D‐2HG production, indicating that resistance requires their co‐occurrence with the primary R140Q driver mutation.

Subsequent studies revealed that second‐site mutations can also occur in *cis*. In a patient with mIDH1 AML treated with ivosidenib, a *cis* S280F mutation in IDH1 prevented ivosidenib binding, conferring drug resistance. Additional second‐site mutations in IDH1 have been identified, including R119P, D279N, G131A, and G289D. These alterations impair binding at the IDH1 dimer interface, disrupting drug‐target interactions and contributing to therapeutic resistance [144]. Currently, no effective strategies exist for overcoming resistance caused by second‐site mutations. However, developing inhibitors that target multiple binding sites or dual IDH1/2 inhibitors may offer strategies to address diverse resistant variants.

Furthermore, two cases of ivosidenib resistance have been described in patients with mIDH1 CCA: One caused by a subsequent D279N mutation in IDH1 and the other by IDH isoform switching [38]. Isoform switching occurs when a tumor driven by an IDH1 mutation acquires dependence on IDH2 (or vice versa), thereby sustaining D‐2HG synthesis and conferring resistance to single‐isoform inhibitors [39]. Studies have shown that in AML patients with the IDH1 R132C mutation treated with ivosidenib, elevated D‐2HG levels correlated with the emergence of an IDH2 R140Q mutation. Similarly, in CCA patients with IDH1 R132C mutations, a de novo IDH2 R172V mutation developed following ivosidenib treatment, resulting in clinical deterioration and death. Another case involved an AML patient with an IDH2 R140Q mutation who achieved long‐term remission with enasidenib but acquired an IDH1 R132C mutation during continued treatment. Resistance driven by isoform switching may be overcome by simultaneous targeting of both IDH1 and IDH2. Preclinical investigations demonstrate that the dual mIDH1/2 inhibitor vorasidenib is effective against resistance attributable to IDH isoform switching [38, 39, 160].

#### 5.1.2 Clonal Selection/Evolution

AML arises from clones of hematopoietic stem/progenitor cells that acquire driver mutations sequentially [161]. Patients harbor multiple clonal subtypes, and drug‐resistant clones are selected during therapy, resulting in treatment resistance [162]. Quek et al. reported that relapses in mIDH2 AML patients treated with enasidenib were driven by clonal selection and evolution [162]. Whole‐exome sequencing and targeted resequencing revealed a broadened mutational spectrum in these patients. Although enasidenib therapy restored normal myeloid differentiation by rebalancing hematopoietic stem, progenitor, precursor, and mature cell populations across all clones, seven distinct patterns of clonal evolution and selection were identified in relapsed patients. These patterns led to a differentiation blockade at transitional clonal stages and eventual resistance. Importantly, clonal evolution and selection did not impair enasidenib binding to the target enzyme, indicating resistance mechanisms independent of target engagement.

Relapses were associated with diverse genetic alterations, including mutations in colony‐stimulating factor 3 receptor and FLT3, loss‐of‐function mutations in the CBL proto‐oncogene, and the emergence of dominant clones with recurrent U2AF1 mutations alongside minor clones harboring the FLT3 D200E variant. Additionally, relapse was linked to mutations in hematopoietic transcription factors, including *RUNX1*, BCL6 corepressor‐like 1 (BCORL1), and *GATA2*, as well as alterations in the BAF chromatin remodeling complex member BCL11A. Chromosome 7 deletions (partial or complete del 7q) were also associated with relapse, with some patients exhibiting clonal selection favoring del 7q clones. Furthermore, less common mutations in genes such as *NFKB1, DDX1, MTUS1, DHX15*, and *DEAF1* contributed to clonal evolution and relapse [163].

The clonal evolution of tumors is a complex and dynamic process that accumulates genetic variations and is subject to selective pressure. The use of the branching process model provides a natural and stochastic evolutionary framework for the birth–death, mutation, and clonal expansion behaviors of tumor cell populations [164]. In past studies, the branching process has been widely employed for theoretical derivations of the probabilities and size distributions of subclones; estimation of the time of emergence of drug‐resistant clones and the level of tumor heterogeneity; and understanding the dynamic mechanisms of drug resistance development from the perspective of mathematical ecology and evolutionary theory. This methodology has been applied to reconstruct the individual evolutionary histories of CLL patients, inferring parameters of cancer evolution [165, 166].

To address these resistance mechanisms, prospective strategies may involve combining enasidenib with additional targeted or chemotherapeutic agents, alongside developing liquid biopsy to prompt early identification of emerging resistant clones. By integrating various types of branching process models with empirical data such as clinical longitudinal sequencing data and dynamic measurements of ctDNA, the prediction accuracy of drug resistance evolution routes can be significantly improved, and quantitative basis for personalized treatment adjustments can be provided [167]. Further research is required to elucidate the molecular mechanisms underlying clonal development and selection to prevent the emergence and dissemination of drug‐resistant clones.

#### 5.1.3 Leukemia Stemness

Emerging evidence indicates that leukemic stemness can lead to primary resistance to mIDH inhibitors in AML [168]. Leukemia stemness refers to the stem cell‐like features of leukemia cells that enable them to self‐renew, undergo multilineage differentiation, and develop drug resistance [169]. LSCs, which represent a distinct subpopulation, play a critical role in leukemia development, relapses, and therapeutic resistance. Gene expression analysis of patients with AML revealed that LSC‐associated genes were elevated in hypermethylated clusters among patients with poor responses to mIDH inhibitors [168]. Forkhead box C1 (FOXC1), a key regulator of LSC function and a driver of the hypermethylated phenotype, was enriched inalong with the LSC‐essential cell surface protein CD99 and the frequently mutated epigenetic regulator DNMT3A. Additionally, a 17‐gene LSC score was generated; multifactorial logistic regression analysis identified enhanced stemness as a major resistance mechanism. Consequently, the stemness score has emerged as a potential predictive biomarker for mIDH inhibitor response. Co‐mutations in genes encoding transcription factors associated with hematological differentiation, such as *RUNX1*, CCAAT enhancer binding protein alpha (CEBPA), and *GATA2*, also influence resistance [168]. Combining mIDH inhibitors with LSC‐targeting agents has shown promise; for example, the addition of BCL‐2 inhibitors, such as venetoclax, eliminates LSCs more effectively than monotherapy [29].

#### 5.1.4 Mitochondrial Metabolism

Abnormal mitochondrial metabolism is extensively studied in cancer due to its association with cell growth, metastasis, and treatment resistance [170]. Oxidative phosphorylation (OXPHOS) is critical for LSC survival and contributes to drug resistance in myeloid leukemia. Stuani et al. reported that mitochondrial metabolic reprogramming in AML confers resistance to mIDH inhibitors [40]. Their multi‐omics and functional research, utilizing mIDH cell lines, PDX models, and AML patient samples, demonstrated that mIDH cells exhibit increased mitochondrial metabolism. In mIDH1 cells, hypermethylation triggers *CEBPA*‐mediated increases in FAO. OXPHOS regulates mitochondrial biogenesis and fatty acid (FA) metabolism, whereas FAO is essential for maintaining OXPHOS and mitochondrial function in AML cells. Although ivosidenib therapy decreased D‐2HG levels and *CEBPA* methylation, it maintained or enhanced mitochondrial activity, including TCA cycle and OXPHOS capacity. Transcriptomic data from patients with mIDH AML revealed a significant increase in OXPHOS gene expression signatures during relapse, suggesting that the maintenance of mitochondrial function may be the primary nongenetic mechanism of resistance. The study also found that combining the OXPHOS inhibitor IACS‐010759 with an mIDH1 inhibitor reduced mitochondrial activity and enhanced efficacy, presenting a novel therapeutic option for patients with resistant AML [40].

#### 5.1.5 MAPK Pathway Activation

Primary resistance to mIDH1/2 inhibitors has been linked to activation of the MAPK signaling pathway. Studies have observed that patients with mIDH2 AML treated with enasidenib harbored activating mutations in numerous MAPK‐related pathway genes, including *NRAS, PTPN11*, serine/arginine‐rich splicing factor 2 (SRSF2), and additional sex comb‐like 1 (ASXL1). The presence of these mutations in enasidenib‐naïve patients suggests that MAPK pathway activation contributes to primary resistance [30]. Mitogen‐activated extracellular signal‐regulated kinase (MEK) is an important component of the MAPK signaling cascade, and it has been a target for combination therapy to overcome drug resistance in other cancers; therefore, the problem of resistance to mIDH inhibitors could be potentially addressed by combining MEK and mIDH inhibitors.

#### 5.1.6 RTK Pathway Activation

Aberrant RTK signaling is associated with the development of various malignancies and resistance to targeted therapies, including mIDH inhibitors. Choe et al. identified RTK pathway mutations as drivers of primary ivosidenib resistance. Comprehensive genomic analysis of patients with mIDH1 relapsed/refractory (R/R) AML revealed that baseline mutations in individual RTK pathway genes (e.g., *NRAS* and *PTPN11*) and grouped RTK pathway genes (including *NRAS, KRAS, PTPN11, KIT*and *FLT3*) were significantly associated with a reduced probability of achieving CR or CRh [31]. This observation aligns with mechanisms underlying enasidenib resistance in AML [30]. Notably, approximately 35% of relapsed patients who initially achieved CR/CRh with ivosidenib monotherapy acquired mutations in the RTK pathway, indicating an association with acquired resistance, although the specific mechanism remains unknown. Other investigations have found that mutations in the RTK pathway can result in acquired resistance to mIDH inhibitors [168]. Although no studies have yet examined the combination of mIDH and RTK inhibitors, this study lays the groundwork for future research into such combinations.

#### 5.1.7 STAT5 Signaling Pathway Conduction

The signal transducer and STAT5 pathway play a critical role in cytokine signaling and are linked to hematopoietic cell survival, proliferation, and differentiation. Liu et al. demonstrated that AML resistance to mIDH inhibitors might be reversed by attenuating STAT5 signaling [32]. Using the murine mIDH1 hematopoietic progenitor cell line OCI‐mIDH1/N, they performed a genome‐wide CRISPR knockout screen to identify genes that, when inactivated, enhance the differentiation response to ivosidenib. The screen identified Clec5a as a target that prevents ivosidenib‐induced differentiation. Clec5a encodes a myeloid cell surface receptor that initiates downstream signaling via splenic tyrosine kinase (SYK) following ligand interaction. Inactivation of Clec5a enhanced ivosidenib sensitivity. Furthermore, blocking SYK signaling with the selective inhibitor R406 improved the differentiation response of WT OCI‐mIDH1/N cells to ivosidenib and decreased their proliferation rate compared to single‐agent therapy. These findings indicate that SYK signaling regulates the differentiation response of mIDH1 cells, primarily by downregulating the stemness‐related *HOX*‐dependent transcriptional pathway. Importantly, STAT5 signaling was found to induce *HOX* gene expression and act as a critical intermediary connecting SYK signaling to the stemness transcriptional pathway. Because mutations in *FLT3, NRAS*, and *PTPN11* activate STAT5, this finding provides a mechanism by which RTK/MAPK mutations enhance resistance: STAT5 serves as a convergence point for upstream SYK and aberrant RTK/MAPK signaling, upregulating stemness‐associated genes. Combining the STAT5 inhibitor pimozide with IDH inhibitors proved more effective than ivosidenib alone [32].

In conclusion, combining mIDH inhibitors with other targeted medications or cytotoxic therapies to overcome monotherapy resistance and improve prognosis has become a popular research topic. mIDH AML cells rely primarily on BCL‐2 for survival [29]. The combination therapy study (NCT03471260) of ivosidenib, venetoclax, and AZA in patients with mIDH1 myeloid malignancies indicated that the triple regimen was well tolerated. The safety profile was similar to that reported for ivosidenib + AZA and AZA + venetoclax. The triple combination dramatically increased survival and resulted in long‐term treatment responses in patients with high‐risk mIDH1 myeloid malignancies [29]. Furthermore, in mIDH CCA, CD8+ T‐cell reduction and loss of tumor cell‐specific TET2 or interferon‐gamma receptor 1 (IFNGR1) lead to mIDH inhibitor resistance. However, blockade of the immune checkpoint cytotoxic T‐lymphocyte antigen‐4 (CTLA‐4) overcomes this immunosuppression, suggesting that synergistic use of immunotherapy can reverse resistance. As the causes of mIDH inhibitor resistance are further elucidated and additional resistance‐associated genes are identified, novel combination therapies offer promising options for patients with refractory or relapsed mIDH1/2 inhibitor monotherapy.

#### 5.1.8 Immune‐Mediated Resistance

Although classical resistance mechanisms have largely focused on genetic and signaling adaptations within tumor cells themselves, increasing evidence highlights that immune‐mediated resistance driven by TME components constitutes a critical and under‐appreciated axis of therapy failure. A large amount of evidence indicates that the immunosuppressive TME and the dynamic changes in metabolites released by tumor cells also trigger metabolic competition, thereby suppressing the immune response. Specifically, immunosuppressive cellular populations, cytokine networks, and metabolic rewiring within the TME interact synergistically to blunt antitumor immune responses and facilitate therapeutic escape [171, 172].

In a Phase III clinical trial, nivolumab also failed to improve OS in patients with recurrent GBM compared to the bevacizumab group. The limited efficacy of immune checkpoint inhibitors may be partly attributed to the lack of neoantigens, the ability of glioma cells to evade immune clearance, and the immunosuppressive effect of the TME [173]. A defining feature of the immunosuppressive TME is the accumulation of TAMs, MDSCs, and regulatory T cells (Tregs), which collectively inhibit effector T cell and NK cell functions through cytokine secretion, nutrient depletion, and direct cell–cell interactions. TAMs, particularly those with an M2 phenotype, produce anti‐inflammatory cytokines, growth factors, and matrix‐modifying enzymes that favor tumor persistence while restraining cytotoxic immune responses, significantly contributing to immune‐mediated treatment resistance. Previous studies have already demonstrated the potential regulatory role of CD109 in the expression of PD‐L1 in macrophages. Cui et al. discovered innovatively in intrahepatic CCA mouse models that sCD109 can bind to the macrophage membrane protein FcγRI, thereby activating the FcγRI/SYK/NF‐κB signaling pathway. This confirmed that sCD109 has a positive regulatory effect in promoting the enrichment of CD73+ macrophages in immunologically cold tumors [174]. Loeuillard et al. used an orthotopic murine CCA model to delineate the interaction between immunosuppressive myeloid cells and therapeutic responses to immunotherapy. Notably, targeting TAMs failed to decrease tumor burden. This outcome was likely driven by the compensatory expansion of immunosuppressive granulocytic MDSCs [175]. Moreover, MDSCs have been proven to be capable of enhancing antitumor immune responses; preliminary evidence indicates that the mIDH is associated with a reduction in the number of inhibitory myeloid cells in gliomas. By analyzing transcriptome data and conducting cell experiments on the myeloid cell populations reprogrammed by mIDH, which gained a deeper understanding of gliomas tumors [176, 177]. Collectively, dual targeting of TAMs and MDSCs offers a promising strategy to potentiate the efficacy of ICB.

Beyond cellular components, immune checkpoint signaling is a major contributor to immune‐mediated resistance within TME. Irreversible T‐cell exhaustion has been viewed as contributing to anti‐PD‐1/PD‐L1 resistance, including TIM‐3 and lymphocyte activation gene protein 3 (LAG‐3) will exhibit compensatory effects after treatment, inducing an increase in the expression of other immune surveillance pathways and subsequently enhancing drug resistance [178, 179]. In MHC Class II–positive tumors, anti‐PD‐1 therapy can initially be effective but is followed by upregulation of the immune checkpoint receptor LAG‐3 on TILs [180]. Furthermore, the inhibitory signals produced by the inhibitor TME may nullify the efficacy of PD‐1/PD‐L1 blockade. Preclinical studies have shown that increased TIM‐3 expression on TILs after treatment, which leads to the formation of acquired resistance to PD‐1/PD‐L1 blockade. Wu et al. constructed a genetically engineered mouse model driven by mIDH1. They found that inhibiting the activity of CD8 T‐cells and the inactivation of the tumor cell's autonomous TET2 DNA demethylase revealed that mIDH1 supports CCA tumors through an immune escape program centered on a dual mechanism mediated by 2‐HG [181]. Notably, the establishment of an “immune‐cold” TME has been closely associated with reduced responsiveness to ICB therapy, highlighting the critical role of impaired immune cell recruitment in tumor immune evasion. Emerging studies further suggest that the oncometabolite 2‐HG contributes to this immunosuppressive phenotype by suppressing the expression of the pro‐inflammatory chemokines CXCL9 and CXCL10, thereby impairing CD8^+^ T‐cell recruitment and limiting the infiltration of effector T cells into tumor tissues [85]. To date, the clinical efficacy of chimeric antigen receptor T‐cell (CAR‐T) therapy in solid tumors remains limited. Although antigen loss or downregulation can lead to immune escape and subsequent resistance to CAR‐T therapy in certain settings, accumulating evidence indicates that the immunosuppressive TME constitutes a major barrier to therapeutic success. The TME not only restricts CAR‐T cell infiltration into tumor tissues but also compromises their persistence and effector function [182]. Therefore, developing strategies to overcome TME‐mediated immunosuppression and enhance CAR‐T cell activity has become a critical focus for improving the efficacy of CAR‐T therapy in solid tumors.

### 5.2 Combination Therapies

The clinical efficacy of IDH inhibitors as monotherapy in IDH‐mutant AML and other malignancies has been well‐established; however, response rates remain suboptimal, and disease relapse is common. Recognition that IDH mutations confer distinct molecular vulnerabilities, particularly in epigenetic regulation and DNA repair, has led to the development of rational combination strategies aimed at enhancing therapeutic efficacy, overcoming resistance mechanisms, and improving long‐term patient outcomes.

#### 5.2.1 Mechanistic Rationale for Combination Therapies: Epigenetic and DNA Repair Targeting

Mutations in IDH1 and IDH2 confer neomorphic enzymatic activity that converts α‐KG to the oncometabolite D‐2HG, which accumulates millimolar concentrations within tumor cells and competitively inhibits α‐KG‐dependent dioxygenases. Among the affected enzymes inhibited are the TET family DNA demethylases and Jumonji‐domain histone demethylases, leading to widespread epigenetic reprogramming characterized by the CpG island methylator phenotype. This hypermethylated epigenetic landscape impairs normal cellular differentiation, particularly in hematopoietic stem and progenitor cells in AML and glial precursor cells in gliomas. Together, these alterations create distinct therapeutic vulnerabilities that can be exploited through rational combination strategies [62, 183].

HMAs, such as AZA and decitabine, are nucleoside analogs that are incorporated into DNA and inhibit DNA methyltransferases (DNMTs), resulting in global DNA hypomethylation and reactivation of epigenetically silenced tumor suppressor genes. In IDH‐mutant cancers, where D‐2HG‐mediated inhibition of TET enzymes drives pathological hypermethylation, HMAs provide a complementary epigenetic reversal mechanism that directly counteracts the consequences of mutant IDH activity. When combined with IDH inhibitors, including ivosidenib or enasidenib, this dual epigenetic targeting strategy synergistically reverses the hypermethylated chromatin state, promotes differentiation of malignant cells, and enhances overall therapeutic response. The mechanistic synergy derives from simultaneously reducing D‐2HG production (via IDH inhibition) while actively removing existing aberrant methylation marks (via DNMT inhibition), creating a coordinated epigenetic normalization that neither agent achieves adequately as monotherapy [24, 25].

Beyond epigenetic dysregulation, mIDH confer a distinct DNA repair vulnerability that represents an orthogonal therapeutic target. The oncometabolite D‐2HG impairs the activity of α‐KG–dependent DNA repair enzymes, including ALKBH family demethylases and KDM4A/B histone demethylases, which are critical for the repair of DNA double‐strand breaks and resolution of replication stress [184]. This compromised DNA repair capacity creates a synthetic lethality opportunity with PARP inhibitors such as olaparib, which block the repair of single‐strand DNA breaks through base excision repair pathways. In cells with functional homologous recombination, PARP inhibition leads to manageable single‐strand break accumulation. In contrast, in IDH‐mutant cells with compromised DNA repair machinery, unrepaired single‐strand breaks progress to double‐strand breaks during DNA replication, culminating in replication fork collapse, catastrophic genomic instability, and apoptotic cell death [185]. Preclinical studies have validated this synthetic lethal interaction across multiple IDH‐mutant tumor models, demonstrating that PARP inhibitor sensitivity correlates with D‐2HG concentrations and can be reversed by IDH inhibitor treatment that normalizes DNA repair enzyme function.

Furthermore, combining PARP inhibitors with ATR inhibitors such as AZD6738 amplifies this DNA damage response collapse through complementary mechanisms. Although PARP inhibition prevents single‐strand break repair, ATR inhibition abrogates the replication stress checkpoint that normally allows cells to pause and repair replication‐associated DNA damage. This dual blockade creates intolerable replication stress, specifically in IDH‐mutant cells with baseline DNA repair deficiency, as demonstrated in preclinical studies showing synergistic cytotoxicity in IDH‐mutant models of glioma and CCA that substantially exceeded the effects of either single agent or PARP inhibitor monotherapy [81, 186]. These mechanistic insights establish both epigenetic normalization and synthetic lethality exploitation as foundational principles guiding rational combination therapy development for IDH‐mutant malignancies.

#### 5.2.2 IDH Inhibitors Combined With HMAs

The combination of IDH inhibitors with HMAs represents the most extensively studied and clinically advanced combination strategy in IDH‐mutant AML. The landmark AGILE Phase III trial (NCT03173248) evaluated ivosidenib plus AZA versus placebo plus AZA in newly diagnosed, intensive patient's ineligible to chemotherapy with IDH1‐mutant AML [24, 187]. With extended follow‐up, the combination demonstrated a median OS of 29.3 months compared with 7.9 months in the placebo arm, representing a clinically meaningful improvement. CR rates were 30% in the ivosidenib–AZA arm, with a notably high rate of MRD negativity among responders. The combination was well tolerated, with a safety profile consistent with the known toxicities of each agent and demonstrated faster hematologic recovery than AZA alone [187].

Similarly promising results have been observed with enasidenib–AZA combinations in IDH2‐mutant AML. The AG221‐AML‐005 study, a randomized Phase II trial, compared enasidenib plus AZA‐to‐AZA monotherapy in newly diagnosed patients ≥60 years old who were ineligible for intensive chemotherapy [25]. The combination arm achieved a significantly higher ORR of 74% versus 36% with AZA alone (odds ratio 4.9, *p* < 0.001), with CR rates of 54% and 12%, respectively. Median OS was not reached in the combination arm compared with 11.4 months with AZA monotherapy. Importantly, responders in the combination arm demonstrated more profound reductions in 2‐HG levels and higher rates of IDH2 mutation clearance, suggesting deeper molecular responses [25]. The most common grade ≥3 adverse events included neutropenia, thrombocytopenia, and anemia, with differentiation syndrome occurring in 12% of patients in the combination arm—manageable with corticosteroids and supportive care.

An alternative risk‐adapted strategy was explored in the Beat AML sub‐study, which evaluated enasidenib monotherapy followed by the addition of AZA in nonresponders [188]. In this trial, 60 patients with newly diagnosed IDH2‐mutant AML received enasidenib 100 mg daily for up to five cycles. The composite CR rate (CR + CRi) was 48% with enasidenib monotherapy, with particularly favorable responses in patients harboring the IDH2‐R140 mutation. Among 17 patients who did not achieve CR/CRi with enasidenib alone, the addition of AZA yielded a composite CR rate of 41%, demonstrating that sequential addition of AZA can salvage responses in primary nonresponders. This risk‐adapted approach minimizes unnecessary toxicity while preserving efficacy, offering a personalized treatment strategy for older patients with IDH2‐mutant AML [188].

Single‐arm studies have further corroborated the efficacy of these combinations. A Phase II trial from MD Anderson Cancer Center evaluated enasidenib plus AZA with optional addition of venetoclax or FLT3 inhibitors in 26 patients with IDH2‐mutant AML (7 newly diagnosed, 19 relapsed/refractory) [189]. Among newly diagnosed patients, the composite CR rate was 100%, with all patients achieving MRD negativity by flow cytometry. In the relapsed/refractory cohort, the composite CR rate was 58%, with higher efficacy observed in first relapse than in disease with multiple relapses. Notably, patients receiving the triplet regimen of enasidenib, AZA, and venetoclax demonstrated superior outcomes, with a median OS not reached at median follow‐up of 11.2 months. Exploratory analyses revealed that co‐occurring mutations in KRAS, NRAS, or TP53 were associated with inferior responses, highlighting the importance of mutational context in predicting treatment efficacy [189].

#### 5.2.3 Triplet Regimens: IDH Inhibitors, HMAs, and Venetoclax

Building on the success of dual HMA–IDH inhibitor combinations, recent investigations have explored the addition of venetoclax, a selective BCL‐2 inhibitor, to create triplet regimens with the potential to overcome resistance mechanisms mediated by anti‐apoptotic proteins. A Phase Ib/Ⅱ study evaluated ivosidenib with venetoclax ± AZA in 31 patients with IDH1‐mutant myeloid malignancies [29]. The triplet regimen achieved a composite CR rate of 90%, compared with 83% with the doublet (ivosidenib + venetoclax). Among MRD‐evaluable patients, 63% attained MRD‐negative remissions, and 64% of patients receiving ≥5 treatment cycles achieved IDH1 mutation clearance by digital droplet PCR—a remarkable depth of molecular response. Median event‐free survival and OS were 36 and 42 months, respectively, representing among the longest survival outcomes reported in this patient population [29].

Mechanistic correlates provided critical insights into resistance pathways. Notably, no IDH isoform switching or second‐site IDH1 mutations—known resistance mechanisms to single‐agent ivosidenib—were observed, suggesting that the triplet regimen effectively prevents these canonical escape routes. However, among the nine patients who relapsed, 75% exhibited clonal evolution characterized by acquisition of transcription factor mutations (e.g., RUNX1 and ETV6) or signaling pathway mutations (e.g., NRAS), with relapses predominantly driven by IDH1‐negative clones. Single‐cell DNA sequencing revealed that the triplet regimen successfully cleared IDH1 + NPM1 + NRAS multi‐mutant clones, with relapses arising from preexisting IDH1‐WT subclones. Importantly, CyTOF analysis identified upregulation of the anti‐apoptotic protein MCL‐1 in residual cells from relapsed patients, whereas BCL‐2‐dependent cell populations were effectively eradicated in responding patients, suggesting that MCL‐1 upregulation represents a potential resistance mechanism to venetoclax‐containing regimens [29].

A subsequent pooled analysis of patients from two Phase Ib/2 trials (NCT03471260 and NCT04774393) examined frontline triplet regimens combining HMAs, venetoclax, and either ivosidenib or enasidenib in 60 patients with newly diagnosed IDH‐mutated AML who were ineligible for intensive chemotherapy [190]. The composite CR rate was 92% (55/60), with an ORR of 95% (57/60). Strikingly, 78% of evaluable patients achieved MRD negativity, and the 2‐year OS was 69%, with a cumulative incidence of relapse of only 24%. Early mortality was exceptionally low (2% at 60 days), and the regime was well tolerated with no new safety signals. Notably, patients with treated secondary AML (tsAML) experienced significantly inferior outcomes (2‐year OS 34%), whereas patients without tsAML achieved a 2‐year OS exceeding 80%, underscoring the importance of disease biology in predicting benefit from triplet therapy [190].

#### 5.2.4 Novel Combination Strategies: IDH Inhibitors and Differentiation Agents

Beyond epigenetic modifiers, alternative differentiation‐inducing agents have been explored in combination with IDH inhibitors. ATRA, a well‐established differentiation agent in acute promyelocytic leukemia, has been shown to synergize with enasidenib in IDH2‐mutant AML cells [191]. In vitro studies using TF‐1/R140Q cells and primary AML samples demonstrated that the combination of ATRA and enasidenib (AG‐221) produced superior induction of myeloid differentiation markers (CD14, CD15) compared with either agent alone. Mechanistic investigations revealed that the combination therapy activated autophagy initiation and the RAF‐1/MEK/ERK signaling pathway, both of which were essential for the synergistic differentiation effect. Additionally, the combination therapy reversed aberrant histone H3K9 trimethylation (H3K9me3) levels—an inhibitory epigenetic mark elevated in IDH2‐mutant cells—thereby facilitating chromatin accessibility and transcriptional reactivation of differentiation programs. Importantly, these effects were specific to IDH2‐mutant cells, with no significant activity observed in IDH‐WT cells, suggesting a tumor‐selective mechanism [191]. Although clinical translation of this combination therapy awaits further study, these findings provide proof of concept for differentiation‐based combination strategies beyond HMAs.

#### 5.2.5 Targeting Concurrent Oncogenic Drivers: IDH Inhibitors With FLT3 or JAK2 Inhibitors

mIDH frequently co‐occur with other oncogenic drivers, particularly FLT3 internal tandem duplications (FLT3‐ITD) and JAK2 mutations in myeloproliferative neoplasms (MPNs). A preclinical study examined the efficacy of combining enasidenib (AG‐221) with the FLT3 inhibitor AC220 (quizartinib) in murine models harboring concurrent IDH2‐R140Q and FLT3‐mIDH [186]. Although enasidenib monotherapy induced partial differentiation and reversed DNA hypermethylation, it failed to reduce leukemic burden or significantly prolong survival. In contrast, the combination of enasidenib with AC220 produced synergistic antileukemic effects, including profound reduction in blast counts, restoration of normal hematopoiesis, and significantly prolonged survival. Epigenomic profiling revealed that the combination more effectively reversed aberrant DNA methylation at key regulatory loci, including the GATA2, a gene master regulator of hematopoietic differentiation, leading to transcriptional reactivation and enhanced cellular maturation [186]. These findings have direct clinical implications, as several trials are now evaluating IDH‐FLT3 inhibitor combinations in patients with co‐mutated AML.

Similarly, concurrent JAK2 and mIDH drive aggressive MPN blast‐phase transformation. In a genetically engineered mouse model of JAK2V617F/IDH2‐R140Q co‐mutant MPN, dual inhibition with the IDH2 inhibitor AG‐221 and the JAK2 inhibitor ruxolitinib synergistically reversed disease phenotypes, normalized spleen size, corrected metabolic abnormalities, and promoted myeloid differentiation [28]. Importantly, combination therapy reduced the leukemic stem cell compartment more effectively than either agent alone, suggesting that targeting both pathways is necessary to achieve durable disease control in co‐mutant malignancies.

#### 5.2.6 Synthetic Lethality: IDH Inhibitors Combined With PARP Inhibitors

The DNA repair vulnerability conferred by mIDH has prompted the investigation of PARP inhibitor combinations as a synthetic lethality strategy. Preclinical studies demonstrated that IDH‐mutant AML and MDS cells exhibit impaired HR because of 2‐HG‐mediated inhibition of DNA repair enzymes, rendering them hypersensitive to PARP inhibition [185]. In PDX models of IDH1/2‐mutant MDS and AML, treatment with the PARP inhibitor olaparib produced significant antitumor effects, including reduced leukemic burden and prolonged survival, with activity observed even in models resistant to IDH inhibitors. Notably, PARP inhibition was ineffective in IDH‐WT models, confirming the tumor‐selective nature of this synthetic lethal interaction [185].

The PRIME trial, a Phase II study, evaluated the PARP inhibitor talazoparib in patients with IDH1/2‐mutant myeloid malignancies (NCT03974204). Although detailed results are pending, early reports suggest that PARP inhibition is feasible and demonstrates clinical activity in this molecularly defined patient population. Furthermore, the combination of PARP inhibitors with radiotherapy is under investigation in IDH‐mutant gliomas, where radiation therapy induces DNA double‐strand breaks that cannot be efficiently repaired in the setting of 2‐HG‐mediated DNA repair deficiency. Preclinical models have shown that PARP inhibition enhances radiation‐induced cytotoxicity, leading to greater tumor volume reduction than radiotherapy alone, representing a promising strategy for enhancing therapeutic efficacy while minimizing off‐target toxicity [192].

#### 5.2.7 Olutasidenib‐Based Combinations in Relapsed/Refractory Disease

Olutasidenib, a newer generation IDH1 inhibitor with enhanced potency and selectivity, has been evaluated in combination regimens for R/R IDH1‐mutant AML. A multicohort Phase I/2 trial (NCT02719574) assessed olutasidenib (150 mg twice daily) plus AZA in 67 patients with R/R mIDH1‐mutant AML [22]. The composite CR rate was 31%, with a median CR duration of 20.3 months, which is remarkably durable for a heavily pretreated population (median of two prior lines of therapy, 83% with ≥2 prior regimens). The ORR was 51%, and median OS was 12.9 months. Among patients without prior olutasidenib exposure (*n* = 51), the composite CR rate improved to 37%, with an ORR of 59%, suggesting that olutasidenib–AZA retains substantial activity even in patients with prior IDH inhibitor or HMA exposure. Importantly, 64% of patients dependent on transfusion achieving CR/CRh became transfusion‐independent, highlighting meaningful quality‐of‐life benefits. The combination was well tolerated, with differentiation syndrome occurring in 9% of patients—lower than expected given the heavily pretreated population [22].

In a separate Phase II study, olutasidenib was evaluated as monotherapy or in combination with AZA in patients with IDH1‐mutant AML or MDS [158]. Among relapsed/refractory patients, the ORR was 41%–46%, whereas newly diagnosed patients receiving olutasidenib–AZA achieved an ORR of 77%, demonstrating the regimen's efficacy across disease contexts. Hematologic toxicities were the most frequent adverse events, consistent with the known profile of HMAs, and no new safety signals emerged with the combination.

#### 5.2.8 Safety and Toxicity Considerations in Combination Therapies

Although combination therapies enhance antitumor efficacy, they also introduce overlapping toxicities that require careful monitoring and management. When IDH inhibitors are combined with HMAs such as AZA or decitabine, patients commonly experience enhanced myelosuppression, leading to neutropenia, anemia, and thrombocytopenia. This hematologic toxicity increases susceptibility to infections and may necessitate treatment delays or dose modifications. Gastrointestinal disturbances, including nausea, vomiting, and diarrhea, can also be more pronounced. Additionally, there is a heightened risk of differentiation syndrome, particularly in AML, because of the synergistic effect on cell maturation pathways. Differentiation syndrome typically presents fever, dyspnea, pulmonary infiltrates, pleural or pericardial effusions, and peripheral edema and requires prompt recognition and treatment with corticosteroids [25, 141, 187].

In combinations involving IDH inhibitors and PARP inhibitors, overlapping disruption of DNA repair pathways can lead to cumulative genotoxic stress in both tumor and normal cells. This may result in fatigue, exacerbated hematologic toxicity, and, in rare cases, secondary malignancies. When combined with immune checkpoint inhibitors, IDH‐targeted therapies may exacerbate immune‐related adverse events (irAEs), such as colitis, hepatitis, and endocrinopathies, as the immune‐suppressive TME shifts toward heightened immunoreactivity. Furthermore, BCL‐2 inhibitor combinations, such as venetoclax, carry risks of tumor lysis syndrome and prolonged neutropenia, necessitating vigilant monitoring, prophylactic hydration, and dose ramp‐up strategies to mitigate these risks [29, 190].

In gliomas or other solid tumors, combining IDH inhibitors with radiotherapy may enhance radiation sensitivity but also increase neurotoxicity, manifesting as cerebral edema, seizures, or cognitive dysfunction. Skin and mucosal toxicities may be more severe when irradiation targets sensitive tissues. Overall, while these combination strategies enhance antitumor efficacy, they require careful patient selection, close toxicity monitoring, dose modifications as needed, and comprehensive supportive care to manage adverse effects and optimize clinical outcomes.

#### 5.2.9 Summary and Future Directions

The integration of IDH inhibitors with complementary therapeutic agents—including HMAs, BCL‐2 inhibitors, FLT3 or JAK2 inhibitors, differentiation agents, and PARP inhibitors—represents a paradigm shift in the treatment of IDH‐mutant malignancies. These rational combinations exploit the unique molecular vulnerabilities conferred by 2‐HG production, including epigenetic dysregulation and DNA repair deficiency, to achieve deeper and more durable responses than monotherapy. Triplet regimens combining HMAs, venetoclax, and IDH inhibitors have demonstrated particularly impressive efficacy in newly diagnosed AML, with composite CR rates exceeding 90% and MRD negativity rates approaching 80%—outcomes that rival or surpass those achieved with intensive chemotherapy in younger, fit patients [190]. Meanwhile, synthetic lethality approaches with PARP inhibitors and targeted inhibition of concurrent oncogenic drivers offer promising strategies for overcoming resistance and expanding the therapeutic window.

Future research should focus on refining patient selection through integrated genomic and epigenomic profiling to identify those most likely to benefit from specific combinations, developing biomarkers predictive of response and resistance (such as MCL‐1 expression or co‐occurring signaling pathway mutations), and exploring novel quadruplet regimens or sequential strategies that maximize efficacy while minimizing toxicity. Additionally, the extension of these combination principles to IDH‐mutant solid tumors, including gliomas and CCA, holds promise for transforming outcomes in these challenging malignancies. As these combination strategies continue to mature through ongoing clinical trials, they hold the potential to establish new standards of care and significantly improve long‐term survival for patients with IDH‐driven cancers.

### 5.3 Mutant IDH Inhibitors in Immunotherapy

mIDH have been shown to impact tumor immune activity in several studies [85]. The use of mIDH1 inhibitors promoted the recruitment of CD8+ T cells and the in vivo production of IFN‐γ in a genetically modified mouse model, to promote immunoevasion and tumor maintenance in CCA [181]. Clinical trial data demonstrate that in LGGs treated with vorasidenib and ivosidenib, IFN‐γ pathways were upregulated when D‐2HG concentrations were low, accompanied by an increase in CD8+ T‐cell infiltration [193]. mIDH1 CCA suppresses innate immunological signaling by hypermethylating and silencing the cytoplasmic double‐stranded DNA (dsDNA) sensor cGAS. Mutant IDH1 inhibitors not only directly impede the action of the mutant enzyme, but they can also boost tumor immunity by activating the dsDNA‐sensing pathway. Mutant IDH1 inhibitors stimulate immunologically essential dsDNA synthesis and enhance cGAS sensing of dsDNA. The dsDNA generated by transposable element‐reverse transcriptase activates cGAS, causing viral mimicry and boosting antitumor immunity [194].

From an immunological point of view, IDH is a potential target for immunotherapy as a tumor‐specific neo‐antigen. Platten et al. investigated IDH1 vaccines and discovered a naturally occurring immunogenic epitope on IDH1 R132H that could be used for mutation‐specific vaccination [195]. In animal models, IDH1 R132H vaccines stimulated specific antitumor immune responses against IDH1 R132H mutant tumors. IDH1 R132H generated a new epitope on MHC Class II, which triggered CD4+ T helper‐1 (Th1) responses against the mutation. This suggests that IDH1 R132H can be recognized by CD4+ Th1 cells. Natural antibodies were specifically recognized in patients with mIDH1 R132H glioma [95]. Later, Legatti et al. used four short peptides (two 9‐mers and two 10‐mers) to immunize mIDH1 glioma mice. This led to an increase in the number of peripheral CD8+ T cells, an increase in the production of IFN‐γ, and the development of an anti‐mIDH1 antibody, all of which significantly increased survival rates [196]. IDH1 R132H‐specific vaccinations have not been linked to any cases of off‐target harm, and IDH vaccines have shown a good safety profile [197]. These findings suggest that IDH may serve as a therapeutic target for T‐cell‐mediated recognition of mutant epitopes, alongside pharmacological inhibition of enzyme activity [95]. Certain anti‐IDH vaccinations may develop into a unique treatment approach for mIDH1 cancers because mIDH is widespread in tumors.

Several clinical trials for IDH1 peptide vaccines are presently underway. These include the IDH1 peptide vaccine alone (NCT02771301), combined with temozolomide (NCT02193347), and co‐targeted with an immune checkpoint inhibitor targeting PD‐L1 (NCT03893903). NOA‐16 is a Phase I clinical trial for an IDH1 R132H‐specific peptide vaccine (IDH1‐vac) to treat Grades 3 and 4 IDH‐mutant astrocytomas (NCT02454634) [197]. Thirty‐two patients in the trial received immunotherapy and were included in the safety dataset (SDS), but two patients were removed from the immunogenicity analysis because immunogenicity testing was not available. IDH1‐vac‐induced immune responses were found in multiple MHC alleles in 93.3% (28/30) of patients in the immunogenicity dataset (IDS). In SDS, TEAEs were reported in 90.6% of patients, with one patient experiencing severe TEAEs and one patient temporarily discontinuing treatment. Twenty‐one (sclerosis, 95% CI: 46.81–81.43) and 15 (erythema, 95% CI: 29.09–65.26) of the TEAEs potentially related to IDH1‐vac were localized site‐of‐administration conditions, which is expected for subcutaneous peptide/protein vaccines using these adjuvants. The ORR for SDS was 84.4% (95% CI: 67.21–94.72), whereas it was 86.7% for IDS (95% CI: 69.28–96.24). The 3‐year PFS for SDS was 63% (95% CI: 44–77), while it was 82% for IDS (95% CI: 62.3–92.1). T‐cell immune responses generated by IDH1‐vac were observed in 26 of 30 patients, while B‐cell immune responses spanning numerous HLA alleles were observed in 28 of 30 patients. Notably, these responses were independent of the in vitro HLA affinity of the IDH1 R132H peptide. This trial demonstrated the safety and immunogenicity of IDH1‐vac in patients with Grades 3 and 4 mIDH astrocytoma and that the immunogenicity of IDH1‐vac was not reliant on HLA type [197]. In conclusion, both preclinical studies and clinical trials indicate that immunotherapy is safer and efficacious. However, research on IDH vaccines remains in its nascent phases, necessitating further investigations to evaluate their safety and immunogenicity.

## 6 Biomarker‐Guided Therapeutic Monitoring and Response Assessment

**FIGURE 5:**
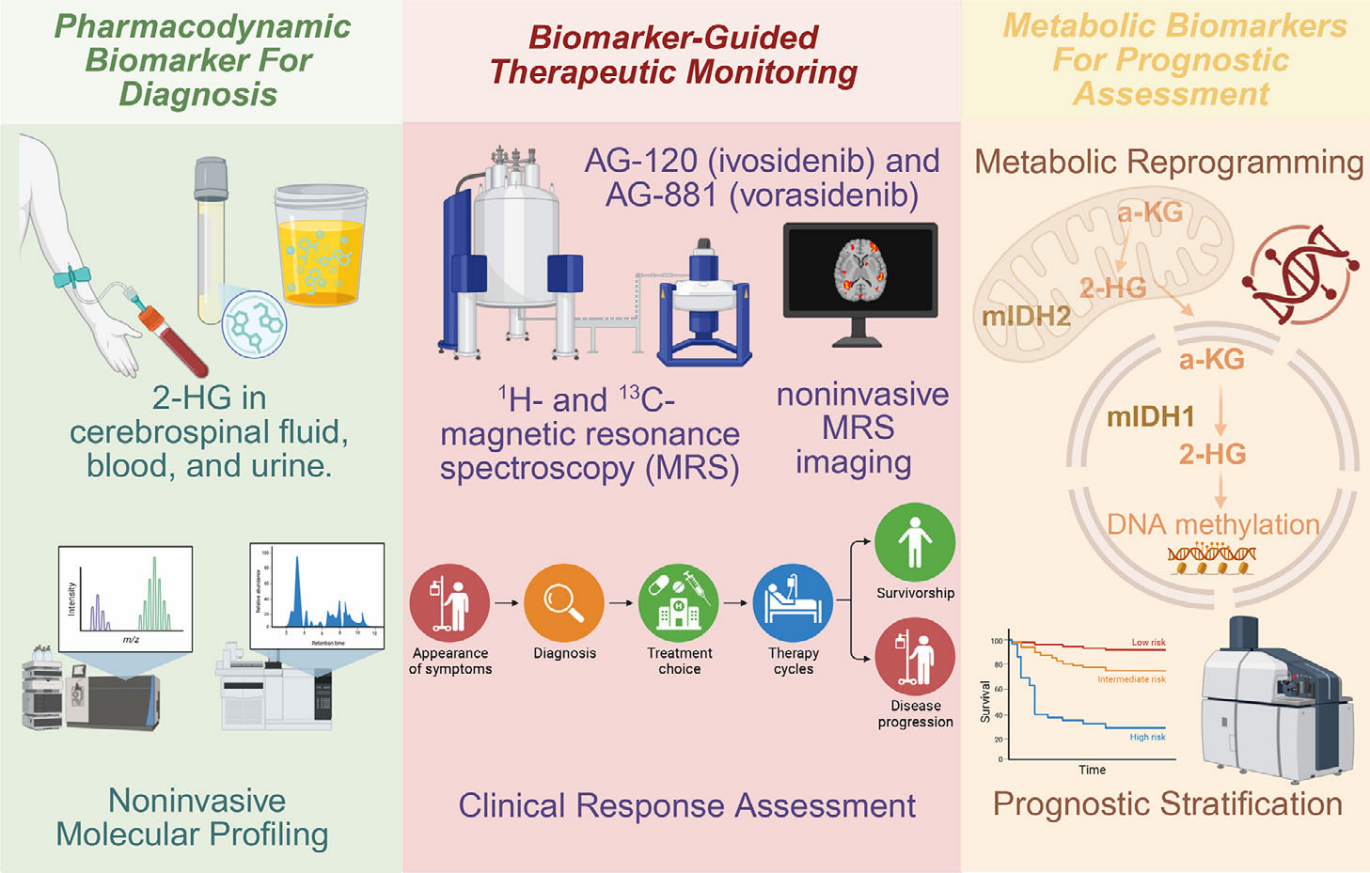
Biomarker‐guided therapeutic monitoring and response assessment. By developing liquid biopsy methods for IDH mutant malignant tumors, including CSF, blood, and urine, with 2‐hydroxyglutaric acid as a guiding therapeutic biomarker for monitoring; and developing a range of detection methods for response assessment, from noninvasive magnetic resonance spectroscopy analysis to including noninvasive MRS imaging, complementary metabolite analysis, and liquid biopsy platforms. mIDH, mutant isocitrate dehydrogenase.

The millimolar accumulation of 2‐HG in IDH‐mutant malignancies provides a unique pharmacodynamic biomarker for diagnosis, treatment monitoring, and prognostic stratification. As the direct enzymatic product of mutant IDH activity, 2‐HG concentrations dynamically reflect target engagement during therapy, positioning this metabolite as an ideal endpoint for assessing treatment efficacy and detecting emerging resistance. The development of diverse detection methodologies—ranging from noninvasive magnetic resonance spectroscopy (MRS) to liquid biopsy platforms—has supported the clinical translation of metabolic biomarkers across key stages of disease management, including initial diagnosis, treatment monitoring, and recurrence surveillance, with further optimization needed to fully realize their potential in routine clinical practice (Figure 5).

### 6.1 Diagnostic Applications and Molecular Subtyping

2‐HG accumulates to millimolar concentrations (5–35 mM) in IDH‐mutant tumors, representing a 100‐fold elevation compared with WT tissues and establishing it as a highly specific biomarker [198]. Pioneering studies by Andronesi et al. demonstrated that optimized MRS techniques—including 2D LASER‐COSY and 1D MEGA‐LASER sequences—can noninvasively detect 2‐HG in vivo with high specificity, thereby successfully distinguishing the characteristic D‐2HG cross‐peak (4.02/1.91 ppm) from overlapping glutamate and glutamine resonances [199]. Using 3 T clinical MR systems, Pope et al. achieved 100% sensitivity in detecting 2‐HG in mIDH gliomas, although a 26% false‐positive rate occurred in WT samples because of spectral overlap, with Cramer–Rao lower bounds (CRLB) serving as quality indicators to distinguish true positives from artifacts [198].

#### 6.1.1 2‐HG as a Specific Biomarker for mIDH

The diagnostic threshold of 2‐HG >1.0 mM demonstrated 100% sensitivity and 88% specificity for identifying IDH‐mutant gliomas in a prospective 136‐patient cohort, with concentrations strongly correlating with tumor cellularity (Spearman *r* = 0.90) and histological grade [200]. Complementary biomarkers further enhance diagnostic accuracy: combined assessment of 2‐HG (>1.8 mM) and glutamate (<3.9 mM) increased area under the ROC curve from 0.79 (2‐HG alone) to 0.92, improving sensitivity from 56% to 72% while maintaining 96% specificity [201]. This glutamate depletion in IDH‐mutant tumors reflects metabolic reprogramming through upregulated glutamate dehydrogenase (GDH1/2) and downregulated BCAT1 expression in LGGs; this dual‐marker approach achieved 88% sensitivity and 100% specificity (AUC 0.98), effectively eliminating false negatives. Additional metabolic alterations include elevated choline (*p* = 0.01) and depleted glutathione (*p* = 0.03) in mIDH tumors, with 2‐HG concentrations positively correlating with Ki‐67 proliferation indices (*r* ^2^ = 0.59, *p* = 0.026) [198].

#### 6.1.2 Liquid Biopsy Technologies for Clinical Translation

Detection of circulating 2‐HG in peripheral blood offers minimally invasive diagnostic potential, although technical challenges exist. In IDH‐mutant CCA, the ratio of circulating R‐2HG/S‐2HG (rRS) demonstrated superior diagnostic performance, with rRS > 1.5 achieving 90% sensitivity and 96.8% specificity [202]. However, plasma 2‐HG levels alone showed limited discriminatory power in patients with glioma, likely reflecting BBB constraints. Enhanced diagnostic accuracy was achieved through combined plasma and urine assessments, with the plasma‐to‐urine 2‐HG ratio (Ratio_2HG) yielding AUC 0.83 in HGG, demonstrating 76% sensitivity and 89% specificity at a cutoff of 20 [203]. Urinary 2‐HG measurement showed modest diagnostic utility (AUC 0.684) on its own [204]. Gas chromatography–mass spectrometry (GC–MS) methods enable routine quantification with sensitivity reaching 10 ng/mL [205].

CSF represents the most informative liquid biopsy compartment for gliomas, with 2‐HG concentrations directly reflecting tumor burden. In a cohort with 30 WT controls, levels of 2‐HG in CSF significantly decreased following tumor resection and chemoradiotherapy, with D/L‐2HG ratios enabling dynamic monitoring of treatment response and recurrence detection [206]. Combined CSF assessment of D‐2HG and circulating tumor DNA (ctDNA) via droplet digital PCR achieved an AUC of 0.7376 for distinguishing IDH‐mutants from WT gliomas, with ctDNA detection providing 100% specificity and D‐2HG correlating with tumor volume [207]. CSF‐based enantioselective detection methods specifically quantify D‐2HG, the pathogenic stereoisomer produced by mutant IDH enzymes [208].

Urinary 2‐HG measurement offers the least invasive liquid biopsy approach, with patients with mIDH glioma demonstrating elevated 2‐HG/creatinine ratios compared with WT cases. Although urine 2‐HG alone showed modest diagnostic performance (AUC 0.684), integration with plasma measurements substantially improved accuracy [203, 204].

#### 6.1.3 Diagnostic Performance and Clinical Utility

The clinical integration of the 2‐HG biomarker assessment addresses critical diagnostic gaps. In 18 patients with glioma deemed surgically high risk and lacking tissue diagnosis, 2‐HG >1 mM threshold enabled presumptive IDH‐mutant classification in 67% (12/18) of cases, with 2‐HG concentration distributions statistically indistinguishable from histologically confirmed cohorts (*p* = 0.64) [200]. This noninvasive molecular profiling proves particularly valuable given that IDH1 R132H immunohistochemistry fails to detect 15% of mIDH, and genetic sequencing requires tissue procurement with associated morbidity. Furthermore, 2‐HG MRS demonstrates excellent test–retest reliability (*r* = 0.98, variance ±0.3 mM), establishing it as a reproducible biomarker for longitudinal monitoring [200]. The technique guides neurosurgical planning by identifying metabolically active tumor regions, optimizing biopsy targeting to enhance diagnostic yield while minimizing sampling error from tumor heterogeneity.

### 6.2 Treatment Monitoring and Pharmacodynamic Assessment

#### 6.2.1 Monitoring IDH Inhibitor Efficacy

2‐HG serves as an ideal pharmacodynamic biomarker for IDH‐targeted therapies, directly reflecting on‐target enzyme inhibition. In an 8‐patient cohort receiving the IDH1 inhibitor IDH305, 3D multivoxel MR spectroscopic imaging (MRSI) detected 70% reduction in tumor 2‐HG concentrations within 1 week of treatment initiation, demonstrating rapid target engagement [209]. Preclinical validation using AG‐120 (ivosidenib) and AG‐881 (vorasidenib) in IDH‐mutant glioma models confirmed dose‐dependent 2‐HG suppression via both ^1^H‐MRS and ^13^C‐MRS isotope tracing, accompanied by reciprocal glutamate elevation reflecting restoration of normal IDH enzymatic function [210]. The noninvasive nature of MRS‐based monitoring enables serial assessments without repeated biopsies, addressing a critical limitation in CNS malignancies where surgical resampling carries a substantial risk. Additionally, PI3K/mTOR pathway inhibition with XL765 demonstrated secondary 2‐HG reduction in IDH‐mutant LGGs, indicating that 2‐HG levels can report on the efficacy of agents targeting downstream oncogenic pathways [211].

#### 6.2.2 Conventional Chemoradiotherapy Response Assessment

Longitudinal 2‐HG quantification provides objective metrics for evaluating chemoradiotherapy efficacy in IDH‐mutant gliomas. In 25 patients undergoing standard‐of‐care treatment, 3D MRSI revealed significant 2‐HG reductions (median 48.17% decrease) following combined radiotherapy and temozolomide, with the magnitude of 2‐HG suppression demonstrating linear correlation with Karnofsky performance status improvement [212]. Temporal dynamics further inform treatment monitoring: during the indolent disease phase (median follow‐up: 18 months), 2‐HG concentrations remained stable with fluctuations of <±1 mM, whereas progression was heralded by exponential 2‐HG elevation 2–6 months prior to radiographic changes, with concentrations rising from baseline 2.42 mM to a maximum of 6.59 mM at progression [200]. Importantly, 1p/19q co‐deleted oligodendrogliomas exhibited faster treatment responses than astrocytomas, with 2‐HG decay time constants of 3.3 versus 11.0 months (*p* = 0.01), despite equivalent volumetric tumor reductions [200].

Complementary metabolic markers enhance response assessment: glutamate/glutamine elevation detected via ^13^C‐MRS constitutes an early indicator of temozolomide efficacy, preceding anatomical changes [213]. Posttreatment surveillance demonstrated sustained low 2‐HG levels (1.9 ± 0.3 mM) in remission, with recurrence detected by rising concentrations (e.g., 1.2–4.4 mM at 35 months) coinciding with new gadolinium enhancement [200].

#### 6.2.3 Clinical Significance of Dynamic Monitoring

The prospective 136‐patient longitudinal study by Choi et al. established 2‐HG MRS as clinically actionable across the entire disease continuum—diagnosis, surveillance, progression detection, treatment monitoring, and post‐therapy follow‐up [200]. Serial 2‐HG measurements distinguished five disease states: newly diagnosed (establishing molecular subtype), indolent (confirming SD with concentration variations <1 mM), progressive (detecting exponential increases before imaging), on‐treatment (assessing therapeutic response), and posttreatment surveillance (monitoring for recurrence). This dynamic biomarker approach enables adaptive treatment strategies, potentially triggering intervention at metabolic progression rather than awaiting radiographic confirmation, thereby optimizing therapeutic windows.

### 6.3 Prognostic Assessment and Tumor Biology

#### 6.3.1 Metabolic Biomarkers and Prognostic Stratification

Beyond diagnostic and monitoring applications, 2‐HG and associated metabolic markers provide prognostic stratification within IDH‐mutant glioma cohorts. Higher grade tumors such as anaplastic oligodendroglioma and secondary glioblastoma demonstrate significantly elevated 2‐HG concentrations compared with lower grade lesions (*p* < 0.05), reflecting increased tumor cellularity [200]. Comprehensive tissue metabolomic profiling of 126 clinical specimens identified independent prognostic metabolites: elevated 2‐HG, acetate, and inositol levels correlated with inferior PFS and OS, with combined 2‐HG and inositol‐based patient stratification yielding optimal PFS/OS discrimination [214]. Mechanistically, high expression of cystathionine β‐synthase—a key transculturation pathway enzyme—emerged as a favorable prognostic biomarker in IDH‐mutant gliomas, suggesting metabolic vulnerabilities amenable to therapeutic targeting [215].

#### 6.3.2 Tumor Progression and Recurrence Prediction

The temporal kinetics of 2‐HG elevation enable detection of early progression with lead‐time advantages over conventional imaging. Multivoxel MRSI mapping revealed that 2‐HG concentrations increase globally across tumor volumes during progression, rather than demonstrating focal hotspots, indicating diffuse metabolic activation preceding anatomical expansion [200]. Histopathological validation confirmed that rising 2‐HG levels correspond to increased tumor cellularity and Ki‐67 proliferation indices, mechanistically linking metabolic biomarker changes to underlying tumor biology. In the posttreatment surveillance setting, 14 of 15 patients (93%) maintained 2‐HG ≤2 mM during sustained remission (median 31 months), with the single relapse case demonstrating 2‐HG resurgence (1.2 → 4.4 mM), concurrent with new enhancement [200].

#### 6.3.3 Metabolic Reprogramming and Tumor Heterogeneity

mIDH fundamentally reprogram cellular metabolism beyond 2‐HG accumulation. High‐resolution magic angle spinning spectroscopy of 126 glioma specimens revealed systematic alterations in amino acid, lipid, and phospholipid metabolism distinguishing IDH‐mutant from WT tumors [216]. PDX metabolomic profiling further demonstrated mutation‐specific metabolic phenotypes, with distinct patterns differentiating IDH1 versus IDH2 mutations in chondrosarcoma models [217]. Engineered cell systems confirmed that 2‐HG treatment recapitulates key metabolic features of IDH‐mutant gliomas, including *N*‐acetylaspartate (NAA/NAAG) depletion [218]. Spatial heterogeneity assessment via 3D MRSI revealed intratumoral 2‐HG concentration gradients, reflecting regional variations in mutant cell density and metabolic activity that inform biopsy targeting and focal therapy delivery [212].

### 6.4 MRS Technical Methodology

#### 6.4.1 MRS Technical Evolution (1D → 2D → 3D)

Technical refinements have progressively enhanced 2‐HG detection capabilities. Single‐voxel 1D approaches include short‐echo PRESS sequences (TE = 30–35 ms) providing comprehensive metabolite profiles but suffering from spectral overlap‐induced false positives (∼22%). Long‐echo methods (TE = 97 ms) exploit scalar coupling modulation to enhance 2‐HG signals while suppressing overlapping resonances [198, 200, 219]. Spectral‐editing techniques, particularly MEGA‐LASER and MEGA‐PRESS (TE = 68 ms), eliminate overlapping signals through selective radiofrequency pulses, achieving unambiguous 2‐HG identification at 4.02 ppm without requiring spectral fitting [199, 219]. Two‐dimensional methods, including LASER‐COSY and TOBSY sequences, separate metabolites into cross‐peak patterns in a second spectral dimension, with 2‐HG's Hα‐Hβ cross‐peaks (4.02/1.91 and 4.02/2.24 ppm) uniquely positioned away from other metabolites, providing definitive assignment, albeit with longer acquisition times (∼13 min) [199].

3D MRSI extends single‐voxel approaches to volumetric mapping, enabling assessment of spatial heterogeneity and tumor burden across entire lesions. The 3D fast spectroscopic imaging sequence acquires metabolic data from supravolumes encompassing the tumor and surrounding brain, facilitating comprehensive evaluation of 2‐HG distribution, treatment response heterogeneity, and progression patterns [209, 212].

#### 6.4.2 Advantages of Ultra‐High‐Field MRS

Ultra‐high‐field systems (≥7 T) provide transformative improvements in spectral resolution and signal‐to‐noise ratio. At 7 T, optimized semi‐LASER sequences with TE = 110 ms amplify 2‐HG signals by 2.9‐fold (simulated) to 1.5‐fold (measured) compared with conventional echo times, reducing CRLB values from 8% to 4% and enabling reliable quantification with as few as four signal averages (∼20 s acquisition) [220]. Critically, 7 T MRS differentiates mIDH subtypes: IDH2 R172K mutations generated significantly higher 2‐HG concentrations (9.06 ± 0.87 µmol/g) than IDH1 R132H mutations (2.53 ± 0.75 µmol/g), consistent with enhanced enzymatic efficiency of mitochondrial versus cytoplasmic mutant IDH isoforms [220]. Unsupervised metabolomic analysis of 7 T spectra successfully clustered IDH1‐mutant, IDH2‐mutant, and WT tumors based on distinctive 2‐HG signatures.

#### 6.4.3 Quantitative Analysis and Standardization

Rigorous quantification employs LCModel software incorporating simulated basis sets (generated via GAMMA/PyGAMMA libraries) containing 21 metabolites, including 2‐HG, glutamate, glutamine, and macromolecules [200, 220]. Quality control utilizes CRLB thresholds, with CRLB <20% indicating reliable quantification, while elevated CRLB values (>25%) identify potential false positives requiring exclusion [198]. Water‐referencing normalizes metabolite concentrations to tissue water content, although tumor‐associated edema may confound absolute quantification. Metabolite ratios (e.g., 2‐HG/glutamate + glutamine >1.0) provide internal standardization, reducing variability from acquisition parameters [199]. Test–retest studies confirm excellent reproducibility (*r* = 0.98) when standardized protocols are employed [200].

#### 6.4.4 Technical Challenges and Solutions

Despite advances, several limitations persist. Spectral overlap between 2‐HG (2.25 ppm) and glutamate/glutamine complicates quantification in short‐echo spectra, necessitating advanced fitting algorithms or spectral editing [199]. Chemical shift displacement artifacts in PRESS sequences cause spatial mismatch between voxel locations at different frequencies, potentially sampling non‐tumoral tissue [219]. Motion artifacts and magnetic field inhomogeneities degrade spectral resolution, requiring real‐time correction algorithms and optimized shimming procedures [212]. Low 2‐HG concentrations approaching 1 mM detection limits may yield false negatives in some IDH‐mutant tumors, though multimodal assessment incorporating glutamate depletion mitigates this limitation [201]. Standardization across institutions remains challenging because of variability in acquisition protocols, analysis software, and operator expertise, highlighting the need for consensus guidelines to facilitate clinical implementation.

### 6.5 Multi‐Platform Integration Technologies

Complementary analytical platforms enhance and validate MRS findings. Liquid chromatography–mass spectrometry (LC–MS) and GC–MS serve as gold‐standard methods for 2‐HG quantification in tissue biopsies and biofluids, with detection sensitivities reaching 10 ng/mL [198, 205]. These techniques enable enantiomer‐specific quantification (D‐2HG vs. L‐2HG), confirming that the pathogenic D‐isomer predominates in IDH‐mutant tumors. High‐resolution magic angle spinning NMR spectroscopy analyzes intact 1‐mg tissue specimens, providing comprehensive metabolomic profiles while preserving samples for subsequent genomic and proteomic analyses [199]. Untargeted metabolomics via mass spectrometry platforms identifies global metabolic reprogramming, revealing alterations in amino acid, lipid, and nucleotide metabolism beyond 2‐HG [216]. Integration of ^13^C‐isotope tracing with MRS and mass spectrometry elucidates metabolic flux changes, demonstrating real‐time pathway activity in vivo. Future directions include development of hyperpolarized ^13^C‐pyruvate MRS for assessing IDH enzyme activity dynamically, artificial intelligence‐enhanced spectral analysis for automated quantification, and integration of metabolic imaging with radiomics to generate comprehensive tumor phenotypes that guide precision therapeutic strategies.

## 7 Translational Challenges and Future Opportunities

The successful development of mutant IDH inhibitors, from their initial mechanistic discovery in 2008 to FDA approval of four clinically validated agents, exemplifies the power of precision oncology to translate molecular insights into therapeutic interventions. These achievements have transformed IDH‐mutant malignancies from biologically intriguing but therapeutically orphaned diseases into actionable clinical entities with demonstrable benefits in inducing differentiation and prolonging survival. However, the transition from proof‐of‐concept efficacy to durable clinical impact has illuminated substantial challenges spanning biological complexity, technological limitations, and healthcare system implementation. Emerging technologies—including single‐cell multi‐omics, spatial transcriptomics, and machine learning‐driven analytics—coupled with a deeper understanding of resistance and immune evasion, present unprecedented opportunities to surmount current barriers. This section synthesizes the major translational obstacles confronting IDH‐targeted therapy and delineates enabling strategies poised to unlock next‐generation therapeutic advances.

### 7.1 Overcoming Resistance and Optimizing Combination Strategies

A fundamental challenge confronting IDH‐targeted therapy is intratumoral heterogeneity and the emergence of resistance. As detailed in Section 5, resistance mechanisms include second‐site mIDH, isoform switching between IDH1 and IDH2, clonal selection favoring preexisting resistant subclones, and activation of bypass signaling pathways such as MAPK, RTK, and STAT5 [30, 32, 37, 39]. The clinical observation that responses to IDH inhibitors are often partial and transient underscores the selective pressure exerted by these agents, driving clonal evolution. Addressing this challenge requires moving beyond static tumor profiling toward dynamic, longitudinal monitoring of clonal architecture. Single cell sequencing technologies offer transformative potential by enabling high‐resolution mapping of clonal evolution during treatment. Single‐cell DNA sequencing can track the emergence of resistant mutations, while single‐cell RNA sequencing reveals transcriptional states associated with therapy evasion, such as LSC signatures or metabolic reprogramming [162, 168, 221]. Integrating single‐cell multi‐omics with spatial transcriptomics could help to identify microenvironmental dependencies and vulnerabilities in resistant subclones, guiding the development of adaptive treatment strategies [222, 223].

Although monotherapy has established proof‐of‐concept, the future lies in rationally designed combinations that address resistance and deepen response quality. Preclinical and early clinical evidence supports combining IDH inhibitors with HMAs, venetoclax, or PARP inhibitors, leveraging synergistic mechanisms including epigenetic reprogramming, BCL‐2 dependency, and synthetic lethality [24, 185, 224, 225]. Triple‐combination regimens, for example, IDH inhibitor + HMA + venetoclax have achieved complete response rates (CRRs) exceeding 90% in AML, representing a substantial advance over monotherapy [29]. However, critical questions remain regarding optimal sequencing and scheduling of combinations, as timing may significantly impact efficacy. Long‐term durability of combination responses and MRD eradication requires prospective validation. Moreover, predictive biomarkers to guide patient selection for specific combinations are urgently needed. Although BCL‐2 dependency predicts venetoclax sensitivity, similar companion diagnostics for other combinations are lacking. Basket and umbrella trials that stratify patients by molecular features rather than histology could efficiently identify efficacy signals across cancer types and accelerate biomarker discovery.

Beyond established combinations, integrating IDH inhibitors with immunotherapies holds promise. As discussed in Section 2.2, mutant IDH creates an immunosuppressive microenvironment through D‐2HG‐mediated impairment of T‐cell function, NK cell cytotoxicity, and DC maturation [27, 87]. Reversing this immunosuppression could enhance responses to ICB, therapeutic vaccines, or adoptive cell therapies. Early trials combining enasidenib with immune checkpoint inhibitors have shown preliminary efficacy signals [226] (NCT05484622), but comprehensive studies with optimized dose scheduling and patient selection are needed to fully realize this therapeutic potential. Furthermore, neoantigen‐targeting vaccines exploiting the IDH1‐R132H mutation as a tumor‐specific epitope represent an innovative approach that has demonstrated immunogenicity in early clinical trials, warranting further investigation in combination with IDH inhibitors to potentially transform the immunologically “cold” microenvironment into an immune‐permissive state [195].

### 7.2 Biomarker Development, Technological Innovations, and Clinical Implementation

Despite progress in 2‐HG detection as a pharmacodynamic biomarker, significant gaps remain in biomarker development and clinical implementation. Although MRS demonstrates high sensitivity and specificity for D‐2HG detection in gliomas [199], widespread adoption is limited by technical expertise requirements and interinstitutional variability. Standardizing MRS protocols, developing automated analysis pipelines, and validating cutoff thresholds across diverse populations are essential steps forward. Similarly, liquid biopsy approaches, including plasma and CSF 2‐HG measurements, hold promise for noninvasive monitoring but require rigorous prospective validation [203]. Beyond pharmacodynamic monitoring, predictive biomarkers that identify patients who will benefit most from IDH inhibitors or specific combinations represent a critical unmet need. Emerging evidence suggests that baseline epigenetic states, such as DNA methylation patterns or histone modification signatures, may predict sensitivity to IDH inhibition [227]. Multi‐omic profiling encompasses genomics, transcriptomics, epigenomics, and metabolomics could generate comprehensive molecular portraits for patient stratification. Machine learning algorithms applied to these high‐dimensional datasets may uncover complex biomarker signatures that outperform single‐gene markers. Furthermore, MRD assays using next‐generation sequencing or digital PCR to detect mIDH in peripheral blood or CSF could identify patients with high‐risk before clinical relapse, enabling preemptive interventions [228, 229].

The next frontier in IDH research will be driven by cutting‐edge technologies providing unprecedented resolution of metabolic, epigenetic, and immune landscapes. Metabolomic profiling using mass spectrometry can map global metabolic reprogramming and identify vulnerabilities beyond 2‐HG accumulation [229]. Recent studies reveal mIDH that alter glutamine, glutamate, and glutathione metabolism [71], suggesting potential combination targets. Isotope tracing experiments can dissect metabolic flux changes in vivo, identifying actionable nodes in cancer metabolism [230]. Complementing these metabolic insights, epigenomic technologies such as ATAC‐seq, ChIP‐seq, and whole‐genome bisulfite sequencing elucidating how D‐2HG‐mediated hypermethylation reshapes chromatin accessibility and gene expression [230]. Understanding which epigenetic changes are reversible upon IDH inhibition versus irreversible differentiation blocks can inform us whether chromatin remodeling agents or differentiation inducers are needed to enhance therapeutic efficacy. CRISPR‐based genetic screens systematically identify synthetic lethal interactions with mIDH, uncovering novel drug targets in DNA repair pathways, epigenetic regulators, and metabolic enzymes [231, 232]. These technological advances collectively provide a roadmap for rational target identification and combination strategy development.

Successful translation of these scientific advances into clinical practice requires navigating complex regulatory landscapes and demonstrating health‐economic value. The orphan drug designation for several IDH inhibitors reflects relatively low mutation prevalence in individual cancer subtypes, creating challenges in conducting adequately powered trials and justifying pricing. Health technology assessment agencies increasingly demand cost‐effectiveness analyses, quality‐adjusted life‐year gains, and real‐world effectiveness data. Demonstrating long‐term survival benefits, reduced hospitalization rates, or improved quality of life will be critical for securing reimbursement and patient access. Real‐world evidence from electronic health records and patient registries can complement randomized trial data by providing insights into effectiveness and safety in broader, more diverse populations, including older patients and underrepresented ethnic groups. Adaptive trial designs such as platform trials allowing addition or removal of treatment arms based on interim data can efficiently test multiple combinations simultaneously and accelerate development. Next‐generation inhibitors with improved pharmacokinetics or dual IDH1/2 inhibitions such as vorasidenib necessitate head‐to‐head comparative trials to establish superiority over first‐generation agents, ensuring that therapeutic advances translate into measurable improvements in patient outcomes across diverse clinical settings [153].

## 8 Conclusions and Future Perspectives

The discovery of mIDH in 2008 inaugurated a new era in precision oncology, transforming our understanding of how metabolic reprogramming drives tumorigenesis. Over the ensuing years, the field has progressed from mechanistic elucidation—establishing that neomorphic 2‐HG production induces epigenetic dysregulation, differentiation arrest, and immune evasion—to clinical validation with FDA approvals of ivosidenib, enasidenib, olutasidenib, and vorasidenib. These achievements demonstrate that targeting oncometabolites can yield meaningful therapeutic benefits, with IDH inhibitors achieving CR rates of 20%–35% as monotherapy in relapsed/refractory AML and substantially improved outcomes when integrated into rational combination regimens. Triple‐combination strategies incorporating IDH inhibitors with HMAs and venetoclax have achieved composite CRRs exceeding 90%. Similarly, vorasidenib has delivered unprecedented PFS of 27.7 months in IDH‐mutant gliomas, validating the differentiation therapy paradigm across diverse malignancies.

Despite these advances, critical challenges persist. Therapeutic resistance driven by clonal evolution, bypass signaling pathway activation, and intratumoral heterogeneity limits response durability. The absence of validated predictive biomarkers constrains personalized patient selection, whereas an incomplete understanding of resistance mechanisms impedes rational therapeutic optimization. Addressing these limitations will require integrating emerging technologies including single‐cell sequencing, spatial transcriptomics, and machine learning with mechanistic investigation to identify vulnerabilities and guide adaptive interventions.

The path forward demands sustained interdisciplinary collaboration to refine combination strategies, validate predictive biomarkers, and integrate IDH‐targeted therapies with immunomodulatory approaches that reverse 2‐HG‐mediated immune suppression. As the field transitions from proof‐of‐concept to optimization, IDH‐targeted therapy stands ready to evolve from a niche application into a cornerstone of metabolic precision oncology, ultimately improving outcomes for patients across the spectrum of IDH‐mutant malignancies.

## Author Contributions

Conceptualization: Caiming Xu, Xiaoqing Wang, Yuhan Fang, and Kai Luo. Data collection, writing, and manuscript preparation: Yuhan Fang, Xiaoqing Wang, and Kai Luo. Editing: Caiming Xu, Xiaoqing Wang, Tikam Chand Dakal, and Guixin Zhang. Figure preparation: Yuhan Fang, Xiaoqing Wang, and He Bai. Revisions: Yuhan Fang, Kai Luo, and Caiming Xu. Supervision: Caiming Xu and Guixin Zhang. All authors have read and approved the submitted version of the manuscript.

## Funding

This work was supported by the United Foundation for Dalian Institute of Chemical Physics, Chinese Academy of Sciences and the Second Hospital of Dalian Medical University (YGHZ202301), the Dalian Medical University Intensified Program of Wellness Interdisciplinary Research Cooperation Project Team Funding (JCHZ2023004), and the Basic Research Projects of the Liaoning Provincial Department of Education (LJ222510161004).

## Ethics Statement

The authors have nothing to report.

## Conflicts of Interest

The authors declare no conflicts of interest.

## Data Availability

The authors have nothing to report.
